# V_H_ Replacement Footprint Analyzer-I, a Java-Based Computer Program for Analyses of Immunoglobulin Heavy Chain Genes and Potential V_H_ Replacement Products in Human and Mouse

**DOI:** 10.3389/fimmu.2014.00040

**Published:** 2014-02-10

**Authors:** Lin Huang, Miles D. Lange, Zhixin Zhang

**Affiliations:** ^1^Department of Pathology and Microbiology, University of Nebraska Medical Center, Omaha, NE, USA; ^2^Eppley Institute for Research in Cancer, University of Nebraska Medical Center, Omaha, NE, USA

**Keywords:** V_H_ replacement, RAG, B cell, IgH gene, IGH sequencing, VDJ rearrangement

## Abstract

V_H_ replacement occurs through RAG-mediated secondary recombination between a rearranged V_H_ gene and an upstream unrearranged V_H_ gene. Due to the location of the cryptic recombination signal sequence (cRSS, TACTGTG) at the 3′ end of V_H_ gene coding region, a short stretch of nucleotides from the previous rearranged V_H_ gene can be retained in the newly formed V_H_–D_H_ junction as a “footprint” of V_H_ replacement. Such footprints can be used as markers to identify Ig heavy chain (IgH) genes potentially generated through V_H_ replacement. To explore the contribution of V_H_ replacement products to the antibody repertoire, we developed a Java-based computer program, V_H_ replacement footprint analyzer-I (V_H_RFA-I), to analyze published or newly obtained IgH genes from human or mouse. The V_H_RFA-1 program has multiple functional modules: it first uses service provided by the IMGT/V-QUEST program to assign potential V_H_, D_H_, and J_H_ germline genes; then, it searches for V_H_ replacement footprint motifs within the V_H_–D_H_ junction (N1) regions of IgH gene sequences to identify potential V_H_ replacement products; it can also analyze the frequencies of V_H_ replacement products in correlation with publications, keywords, or V_H_, D_H_, and J_H_ gene usages, and mutation status; it can further analyze the amino acid usages encoded by the identified V_H_ replacement footprints. In summary, this program provides a useful computation tool for exploring the biological significance of V_H_ replacement products in human and mouse.

## Introduction

Antibodies are the effective molecules in the adaptive immune system to recognize specific antigens and combat bacterial and viral infections, as well as malignant cells (1). To recognize almost unlimited numbers of antigens, a tremendously diversified repertoire of antibody specificities is generated through V(D)J gene recombination, somatic hypermutation, and class switch recombination (1, 2). V(D)J recombination is catalyzed by the recombination activating gene products (RAG1 and RAG2) that recognize recombination signal sequences (RSS) (3–5). Functional RSS consists of a heptamer (CACTGTG), a nonamer (GGTTTTTGT), and a non-conserved spacer region of 12 or 23 base pairs in between (6, 7). Efficient recombination occurs only between a pair of RSSs with 12- and 23-bp spacers, known as the 12/23 rule (7, 8). During V(D)J recombination, the RAG1 and RAG2 complexes first nick between the heptamer and the coding sequence, leaving a blunt signal end and a hairpin sealed DNA coding end (7–9). The two signal ends are usually fused to form a signal joint and the intergenic region will be released as a circular DNA from the chromosome (7–9). The coding end hairpins will be opened and processed by the Artemis:DNA-PKcs complex (10) and joined by the XRCC4:DNA ligase IV complexes from the non-homologous end joining (NHEJ) DNA repair pathway (7–9). Palindromic nucleotides (P nucleotides) may be generated at the coding ends if the hairpin is nicked off the center (7–9). Non-template nucleotides (N-regions) can be added by the terminal deoxynucleotidyl transferase (TdT), whose expression is restricted to early lymphoid cells during active V(D)J recombination. TdT has a preference for adding G residues, which results in generally GC-rich N-regions (7–9).

Immunoglobulin (Ig) gene V(D)J recombination occurs in a step-wised manner during early B cell development (2, 11, 12). Normally, D_H_ to J_H_ rearrangement occurs before V_H_ to DJ_H_ rearrangement on one of the Ig heavy chain (IgH) alleles, followed by Vκ to Jκ and then Vλ to Jλ rearrangement on the Ig light chain (IgL) loci (2, 11, 12). Due to the random nature of RAG-mediated rearrangements, approximately two thirds of the rearranged Ig genes may be out of the reading frame, which cannot produce functional Ig peptides (13). Functionally rearranged IgH genes may produce IgH peptides that fail to pair with surrogate or functionally rearranged conventional IgL chains (13). Moreover, functional Ig genes may encode self-reactive antibodies (14–16). In order for these B cells to survive, early B lineage cells retain the ability to reinitiate RAG-mediated secondary recombination to alter the rearranged Ig genes, a process known as receptor editing (14–16). Receptor editing of the IgL genes would be easy to envision because the organization of the mouse and human Igκ locus enables continuous secondary recombination by joining an upstream Vκ gene segment with a downstream Jκ gene segment, leading to the deletion of the previously formed VκJκ joint (14, 15). B cells also have a default option to delete the entire Igκ locus and initiate *de novo* rearrangement of the Igλ locus (14, 15). Secondary rearrangement on the IgH locus is conceptually difficult, because the primary rearrangement deletes all D_H_ gene segments flanked by 12-bp RSSs. The remaining upstream V_H_ and downstream J_H_ gene segments are flanked by 23-bp RSSs, which are difficult to recombine (17). Nevertheless, secondary IgH rearrangement to generate functional IgH genes from non-functional IgH rearrangements was observed in mouse pre-B cell lines even before the discovery of the RAG genes (18, 19). Comparison of the non-functional and newly formed functional IgH rearrangements led to the identification of a cryptic RSS (cRSS), TACTGTG motif, embedded at the 3′ end of the rearranged V_H_ genes (18–20). Based on these observations, a novel V_H_ to V_H_DJ_H_ recombination mechanism was proposed as V_H_ replacement (18–20). Subsequent studies demonstrate that V_H_ replacement is employed to rescue pro B cells with two alleles of non-functional IgH rearrangements (17, 21), to edit IgH genes encoding anti-DNA antibodies (22–24), and to change the knocked-in IgH gene encoding monoclonal anti-NP antibodies and to generate a diversified antibody repertoire (25, 26).

V_H_ replacement changes almost the entire V_H_ coding region (27). However, due to the location of the cRSS, a short stretch of nucleotides from the previously rearranged V_H_ gene may be remained at the newly formed V–D junctions after each round of V_H_ replacement (16, 27, 28). Such remnants can be used as footprints to trace the occurrence of V_H_ replacement and to identify potential V_H_ replacement products (16, 27, 28). Our previous analysis of 417 human IgH sequences indicated that V_H_ replacement contributes to the diversification of the primary human antibody repertoire (27). This conclusion was supported or argued by subsequent analyses of IgH genes from human or mouse (29–32). Most of these sequence analyses were based on relatively small number of IgH gene sequences or sequences from few individuals. A comprehensive analysis of large numbers of IgH gene sequences is required to fully address the biological significance of V_H_ replacement in antibody repertoire diversification.

Analysis of Ig gene sequences obtained from B cells of different developmental stages or in different disease states provided tremendous information regarding the development and selection of the antibody repertoire. Currently, there are about 61,000 human and 17,000 mouse IgH gene sequences available at the NCBI database. With the advanced next generation sequencing (NGS) technology, millions of Ig gene sequences can be easily obtained (33–35). To identify potential V_H_ replacement products in a large number of IgH gene sequences and to explore the biological significance of V_H_ replacement products in different diseased subjects in human and mouse, we developed a Java-based computer program, named V_H_ replacement footprint analyzer-I (V_H_RFA-I).

## Materials and Methods

### Computer hardware and software requirements

The V_H_RFA-I program can be operated on any desktop computer with Microsoft Windows, Mac OS X, or different Linux operating system. It requires Java runtime environment (jre) 1.6 or higher version for operating and Microsoft Excel 2007 or higher version for data export.

### Software development

The V_H_RFA-I program was developed using the NetBeans 7.01 IDE with Java development kit (JDK) and tested under Windows, Mac OS X, and Ubuntu Linux. Two free Java libraries were used, a csv parser library^1^ and an Excel parser library^2^.

### Reference human and mouse V_H_ gene sequences

The reference human and moue V_H_ germline gene sequences used for generating the V_H_ replacement footprint libraries were downloaded from the IMGT database and listed in Table S1A,B in Supplementary Material.

### Description of the human and mouse IgH gene sequence training data sets

Two sets of IgH gene sequences, one from human and the other from mouse, were used in the initial testing and training of the V_H_RFA program. The 417 human IgH genes sequences were from a study that examined whether peripheral blood B cells of preterm infants show similar restrictions as fetal liver B cells (36). These sequences had been used in our previous analysis to manually identify potential V_H_ replacement products (27). These sequences are referred as the Z417 test sequences in this study and the results of Z417 test sequences are shown at each step of the analysis.

## Results

### An overview of the V_H_RFA-I program and functional modules

**Figure 1:**
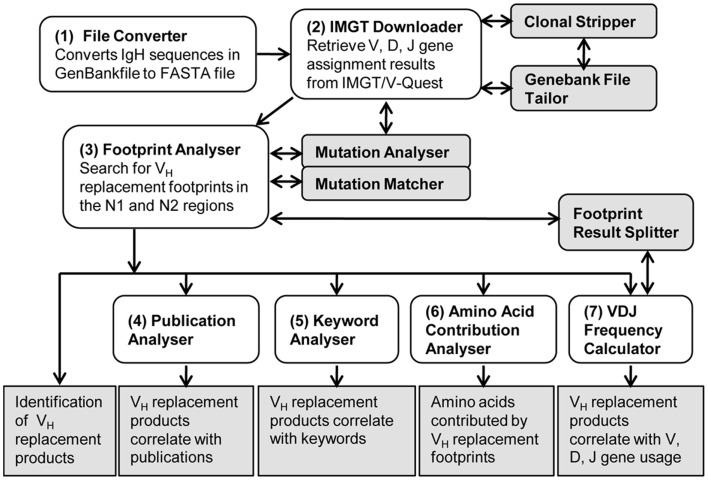
**Overview of the V_H_ replacement footprint analyzer-I (V_H_RFA-I) program**. Diagram shows the workflow of the V_H_RFA-I Program. All the major functional modules are marked with numbers and their functional outcomes are indicated.

**Figure 2:**
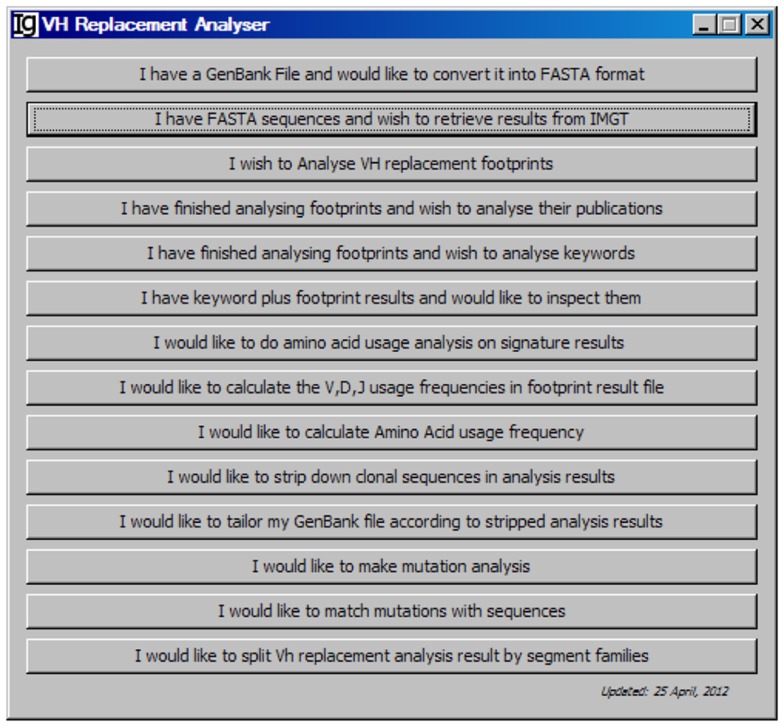
**The front page of the V_H_ replacement footprint analyzer-I (V_H_RFA-I) program**. The V_H_RFA-I program contains multiple functional modules as listed as clickable bars on the front page.

As shown in the workflow of the V_H_RFA-I program (Figure 1), the V_H_RFA-I program consists of multiple functional modules for the analysis of IgH genes and for the identification and analysis of V_H_ replacement products in published or newly generated IgH gene sequences from human or mouse. The V_H_RFA-I program is a single executable Jar file, which can be operated on any computer operating platform. The V_H_RFA-I program can be launched by double click of the executable Jar file, V_H_ Replacement Analyzer-I, which opens the main interface of the V_H_RFA-I program (Figure 2). All the functional modules are listed as clickable bars in the main interface. The detailed functions of these modules are discussed below.

### The FASTA format converter

The *FASTA Format Converter* was designed to convert GenBank files to FASTA files. It can be operated by clicking the first functional bar, *I have a GeneBank File and would like to convert it into FASTA format* (Figure 2). This function module converts IgH gene sequences downloaded from the NCBI database from GenBank format to FASTA format, which can be used for subsequent analysis. This file converter differs from other converters in that it will eliminate entries that do not contain actual sequence data. You can specify the locations of the input GenBank file and the output FASTA file in the pop-up window.

### Retrieve V_H_, D_H_, and J_H_ gene assignment results from IMGT

**Figure 3:**
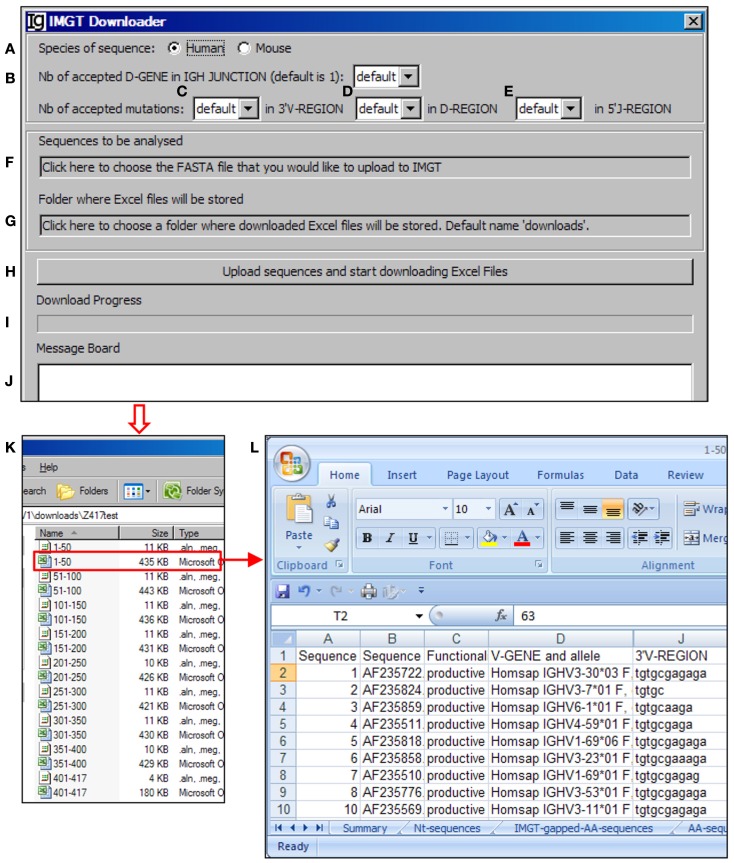
**The IMGT downloader**. Diagram shows the interface of the *IMGT Downloader*. The *IMGT Downloader* allows users to use the IMGT/V-QUEST program to analyze large numbers of IgH gene sequences by uploading IgH sequences and downloading V-QUEST analysis results to a local computer. The user can specify human or mouse sequences **(A)**, numbers of D_H_ genes (default = 1) **(B)**, number of accepted mutations in the 3′ V_H_ region **(C)**, D_H_ region **(D)**, and 5′ J_H_ region **(E)**. After these settings, the user can upload the IgH sequences (in FASTA file) **(F)** and specify the directory where the downloaded V-QUEST analysis Excel files can be stored **(G)**. The analysis can be started by clicking the *Upload sequences and start downloading Excel Files* bar **(H)**. The analysis progress **(I)** and message during the analysis **(J)** will also be shown. The V-QUEST analyses results of the test sequences are downloaded to a user specified location **(K)**. The detailed results of sequence 1–50 are shown in the V-QUEST format **(L)**.

The V_H_RFA-I program uses the IMGT/V-QUEST program to assign the potential V_H_, D_H_, and J_H_ germline genes. In order to handle a large number of IgH gene sequences, we designed the *IMGT Downloader* functional module (Figure 3) to automatically send IgH sequences in batches of 50 sequences in FASTA format to the IMGT/V-QUEST program for analyses^3^ and export the V_H_, D_H_, and J_H_ gene assignment results as Excel files to a user specified local location (Figure 3). The HTTP requests are sent to “http://imgt.org/IMGT_vquest/vquest.” Dependent on the speed of the internet, the V_H_RFA-I program can analyze every 50 IgH sequences within 1 min.

For each analysis, the user can specify the species of IgH sequences (Figure 3A), number of accepted D_H_ germline gene segments (Figure 3B), number of accepted mutations within the 3′ V_H_ gene (Figure 3C), D_H_ gene (Figure 3D), and 5′ of J_H_ gene (Figure 3E). To be analyzed, IgH sequence files can be selected from a local computer and the downloaded result files can be directed to a local computer (Figures 3F,G, respectively). The process will be started after clicking the functional bar: *upload sequences and start downloading Excel Files* (Figure 3H). The downloading process will be indicated in the *Download Progress* window (Figure 3I). If there is any mistake during the file uploading and downloading process, a note will be posted on the *Message Board* (Figure 3J). In the test run of the Z417 test IgH sequences, the V-QUEST analysis results were deposited at a user specified local hard drive with 50 sequences per file (Figure 3K). The results contain all the information from the V-QUEST (Figure 3L). After this step, the downloaded V-QUEST result files can be further analyzed by the V_H_RFA-I program on any local computer.

### Identification of V_H_ replacement footprints

**Figure 4:**
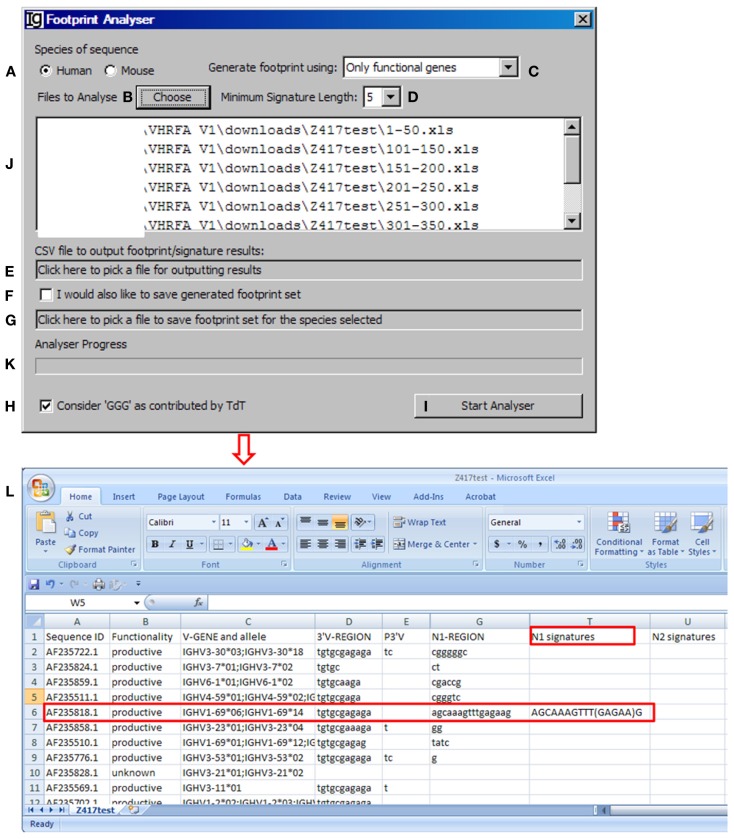
**The footprint analyzer**. Diagram shows the interface of the *Footprint Analyzer*. The user can specify the species of the sequences **(A)**, choose input Excel files downloaded from IMGT/V-QUEST **(B)**, choose the source of footprints used to identify potential V_H_ replacement products **(C)**, set the criterion as to the minimum length of footprints **(D)**, choose the CSV file for storing footprint analysis result **(E)**, chose to store the used footprint file **(F)**, specify the name and location of the used footprint file **(G)**, exclude footprints with “GGG” sequence **(H)**, start the analysis **(I)**. The selected files for analysis will be shown in the window **(J)** (The Z417 test sequences), and analysis progress will be shown in a progress bar **(K)**. The footprint analyses results will be saved in Excel format **(L)**. The identified sequence with 5-mer footprint in the N1 region is highlighted in the red box. The identified footprint (GAGAA) in the N1 region is listed in Column T (N1 signature).

The *footprint analyzer* module uses the sequence analysis results retrieved from the IMGT/V-QUEST program to identify potential V_H_ replacement products. Basically, it searches for potential V_H_ replacement footprint motifs within the N1 and N2 regions of each IgH sequence and export all the analysis results in a single CSV file. The user can specify the species of sequences to be analyzed (Figure 4A, with the Z417 test sequence files), uploaded the files to the program (Figure 4B), select the different V_H_ replacement footprint library (Figure 4C), and specify the minimum length of the V_H_ replacement footprints (Figure 4D).

The *Footprint Generator* functional module is built into the program. It does not have a graphic user interface (GUI) but gets its parameters from and is invoked by the *Footprint Analyzer* (Figure 4C). It loads IMGT germline references (Table S1A,B in Supplementary Material), extracts nucleotide sequences after the cRSS (TACTGTG motif) to generate a library of potential V_H_ replacement footprints with different length. The user has five options to choose the source of the V_H_ replacement footprints library by selecting “only functional genes,” “only non-functional genes,” “all genes,” “functional less non-functional genes,” or “non-functional less functional genes” (Figure 4C). Potential V_H_ replacement footprints for both human and mouse are listed in Table S2 in Supplementary Material, as grouped by lengths. During the primary recombination, the 3′ end of V_H_ genes can be trimmed off by exonuclease activities after processing the coding end hairpin structure. During the V_H_ replacement process, the 5′ end of such footprints could also be trimmed off by exonuclease. *The Footprint generator* can generate a library of potential V_H_ replacement footprints with 3–12 bp in length according to the user’s selection of the *Minimum Signature Length* in the combo box (Figure 4D).

The *Footprint Analyzer* starts to search the longest motifs and then to the shorter motifs based on the user’s selection. The user can specify the location of the output result file (Figure 4E) and also save the footprint library used for each analysis (Figures 4F,G). The analysis progress will be indicated in the *Analyzer Progress* window (Figure 4K). The user also has the option to exclude GGG sequences by checking the checkbox (Figure 4H). The results will be saved in Excel format. As shown in Figure 4L, potential V_H_ replacement footprint with user specified length (5-mer) were identified in both N1 regions (N1 signatures) or N2 regions (N2 signatures) together with the V_H_, D_H_, and J_H_ gene assignment results.

### The publication analyzer

**Figure 5:**
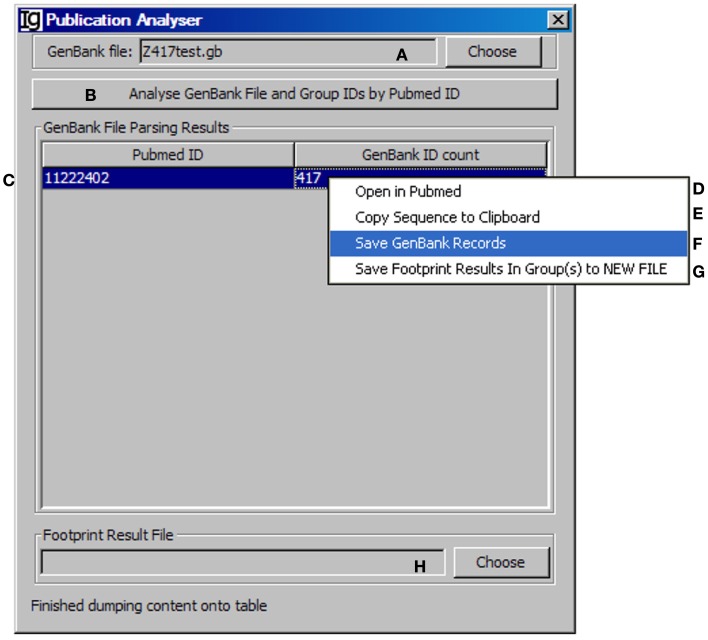
**The publication analyzer**. Diagram shows the interface of the *Publication Analyzer*. The user can choose the input GenBank file **(A)**, start the publication analysis process **(B)**. The number of GenBank records in association with each PubMed ID will be shown in the window area **(C)**. By clicking on each GenBank ID, the abstract pages of selected PubMed IDs at the NCBI database can be opened **(D)**; the GenBank IDs associated with selected PubMed IDs can be copied to the clipboard **(E)**, the GenBank records associated with selected PubMed IDs can be saved **(F)**, or the footprint analysis results associated with selected PubMed IDs can be saved in groups **(G)**. The user can also choose the file containing V_H_ replacement analysis results associated with the GenBank file **(H)**.

All the IgH gene sequences deposited at the NCBI database are linked with their original publications with all the information. To explore the biological significance of the identified V_H_ replacement products, we designed a special *Publication Analyzer* functional module. The *Publication Analyzer* groups IgH sequence analysis results according to their PubMed identifications (PMID). To do so, the user needs to select the original GenBank file (Figure 5A) and the V_H_ replacement analysis results to start the analysis (Figure 5B). In the output results, the V_H_ replacement products results will be linked with the PubMed ID of the original IgH sequence (Figure 5C). Under the GenBank ID pull down manual, the user can open the Abstract pages of selected PubMed IDs (maximum of five) (Figure 5D); copy the GenBank IDs from selected publications to the clipboard (Figure 5E); save GenBank records of selected publications (Figure 5F); and save the V_H_ replacement footprint analysis results of selected publication, as generated by the *Footprint Analyzer* (Figure 5G). The *Publication Analyzer* can also provide the original footprint result file for the selected publications (Figure 5H).

### The keyword analyzer

**Figure 6:**
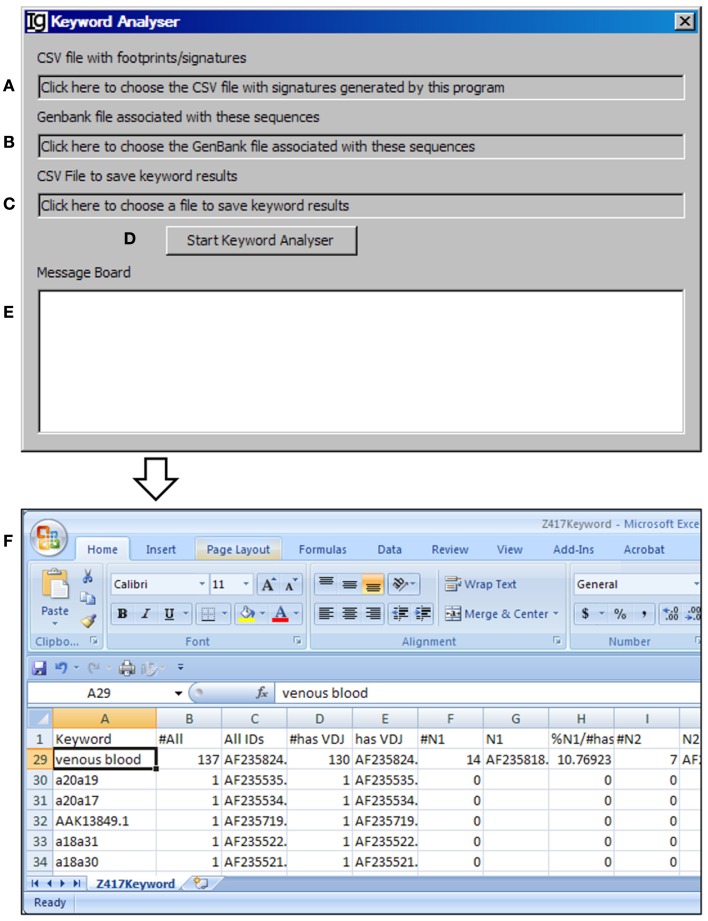
**The keyword analyzer**. Diagram shows the interface of the *Keyword Analyzer*. **(A)** Textbox to choose the V_H_ replacement footprint analysis result file. **(B)** Textbox to choose the GenBank file with the V_H_ replacement footprint analysis result file. **(C)** Textbox to choose the output file. **(D)** Button to start the analysis process. **(E)** Window area to show the message during analysis progress. **(F)** Examples of list of keywords associated with the Z417 test sequences.

The *Keyword Analyzer* groups sequence IDs according to their linked keywords from the GenBank files. The *Keyword Analyzer* will use the footprint analysis result file (Figure 6A), GenBank file containing the original sequences to generate the footprint analysis result file (Figure 6B), keyword analysis result file (Figure 6C). After starting the analysis (Figure 6D), the program will parse the DEFINITION, KEYWORDS, and FEATURES sections of the GenBank record for each IgH gene sequence. An ID will be assigned to a keyword if the GenBank entry contains the keyword. Depending on the availabilities of all VDJ assignments, N1 footprints, or N1 footprints, it also assigns IDs to these bins within each keyword. Same as the *File Format Converter*, the *Keyword Analyzer* ignores GenBank records without actual sequence data. As such analysis takes substantial amount of time when the GenBank file is complex, a log window is provided to monitor the process (Figure 6E). For examples, all the keywords associated with the Z417 test sequences from the NCBI database are listed in Column A, *Keyword* (Figure 6F).

### Assemble the keyword group

**Figure 7:**
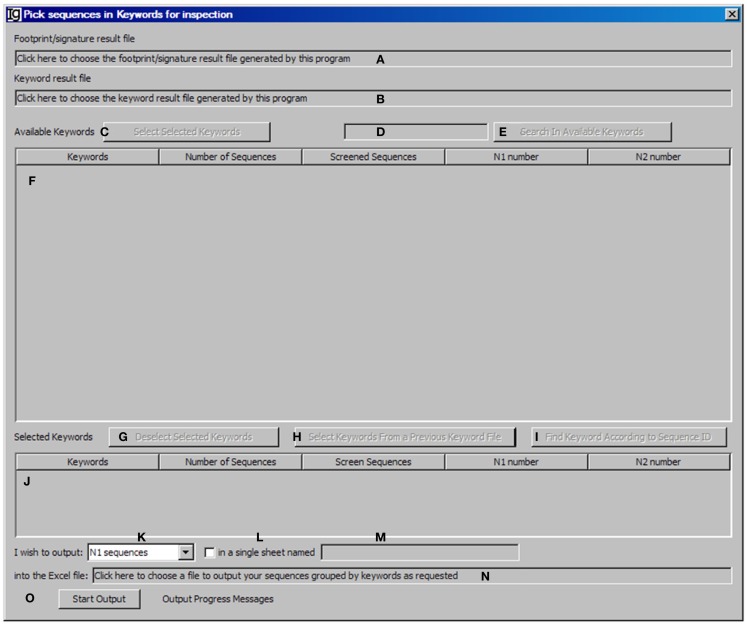
**The keyword group picker**. Diagram shows the interface of *the Keyword Group Picker*. **(A)** Textbox to select the footprint analysis result file. **(B)** Textbox to select the keyword analysis result file. **(C)** Button to move selected rows from **(F)** to **(J)**. **(D)** Textbox for entering search string to locate keywords in **(F)**. **(E)** Button to start locating keywords containing string in **(D)**. **(F)** Window area containing contents of the keyword analysis result file. **(G)** Button to move selected rows from **(J)** to **(F)**. **(H)** Button to select a keyword analysis result file so that keywords can be isolated, to repeat a previous pick. **(I)** Button to select keywords associated with entered GenBank ID. **(J)** Window area displaying the selected keywords. **(K)** Combo box to select the type of sequences to output. **(L)** Checkbox to indicate intention to dump footprint analysis result into a single sheet. **(M)** Textbox for entering the sheet name if **(L)** is selected. **(N)** Textbox for choosing the output file. **(O)** Button to start the pick/isolation process.

The *Keyword Group Picker* visualizes results from keyword analysis and footprint analysis, allowing the user to select group of keywords of interest and output the related footprint analysis results. This functional module analysis provides the user an opportunity to manually inspect a subset of sequences for particular studies. After selecting the footprint analysis result file (Figure 7A) and choosing the keyword analysis result file (Figure 7B), the results ordered by keywords ascending alphabetically and case insensitive will be shown in the table below (Figure 7F). Typing inside the table with the first letter of any keyword will allow quick location of the keywords. The user can also select specific keywords (Figure 7C) to move them from the upper window (Figure 7F) to the lower window (Figure 7J) for further analysis or deselect the keywords (Figure 7G). Pressing *Enter* (Figure 7D) or clicking the functional bar (Figure 7E) will select all keywords containing strings. The user can also select keywords from a picked file (Figure 7H) or select keywords according to their sequence IDs (Figure 7I). The user needs to specify the name and location of the output result file (Figure 7N). There are four options for the output results, which can be specified by the user (Figure 7K): “all sequences” will select footprint analysis results in all the keywords listed in the lower window (Figure 7J); “Screened Sequences” will select those with all V, D, and J assignments; “N1 Sequences” will select those with footprints in the N1 region; “N2 Sequences” will select those with footprints in the N2 region. The format of the output results can also be specified by checking the checkbox (Figure 7L) and providing a name (Figure 7M), in which the results will be exported as an Excel file in which the first sheet contains statistics, the second sheet contains the merged footprint analysis results, and the third sheet contains the results as shown in the lower window (Figure 7J). Otherwise, the footprint analysis results will be exported in separate sheets according to keywords. The analysis can be started by clicking the *Start Output* bar (Figure 7O).

### The amino acid contribution analyzer

**Figure 8:**
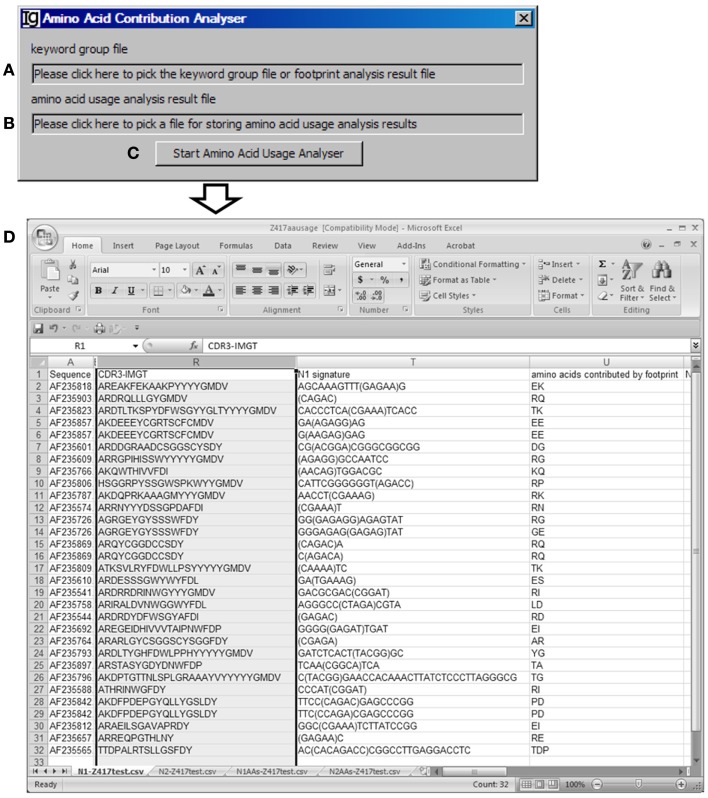
**The amino acid contribution analyzer**. Diagram shows the interface of *the Amino Acid Contribution Analyzer*. **(A)** Textbox for selecting the footprint analysis result file. **(B)** Textbox for selecting the output file. **(C)** Button for starting the analyzer. **(D)** A sample result showing the V_H_ replacement footprints and amino acid residues encoded by the identified V_H_ replacement footprints the test sequences.

The *Amino Acid Contribution Analyzer* analyzes the IgH CDR3 amino acid sequences and identifies the amino acids contributed by the identified V_H_ replacement footprints in the N1 or N2 regions. If the input file is an Excel file, it iterates through all footprint analysis result sheets and generates four sheets: “N1-” sheet contains sequences with N1 footprint; “N2-” sheet contains sequences with N2 footprints; “N1AAs-” contains results with amino acids contributed by N1 regions; “N2AAs-” contains results with amino acids contributed by N2 regions. An amino acid is considered to be contributed by a V_H_ replacement footprint if the first or second nucleotide of its codon is encoded by the footprint. The user can select the *Input Fil*es (Figure 8A) from all the analyzed results, such as Excel files generated by the *Keyword Group Picker*, or CSV files generated by the *Footprint Analyzer*. The user also needs to specify the location of the output file (Figure 8B). The analysis can be started by clicking the “*Start Amino Acid Usage Analyzer*” bar (Figure 8C). As an example, the amino acids contributed by the identified footprints in Z417 test sequences are listed following the N1 signature (Figure 8D).

### The amino acid usage calculator

**Figure 9:**
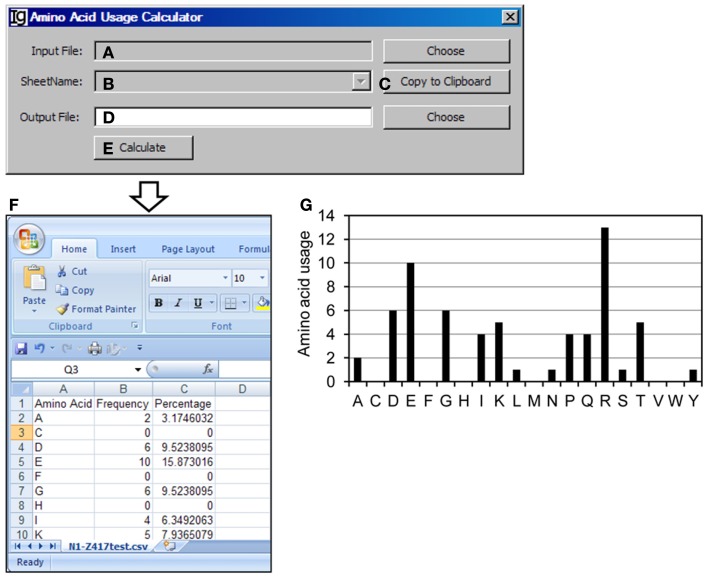
**The amino acid usage calculator**. Diagram shows the interface of *the Amino Acid Usage Calculator*. **(A)** Button to choose the amino acid analysis result file. **(B)** Combo box for choosing the sheet to analyze. **(C)** Button to copy the name of the selected sheet to the clipboard. **(D)** Button to choose the output file. **(E)** Button to start the calculation process. **(F)** The output results of amino acid usage in Excel format. **(G)** Bar graph shows the amino acid usages.

The *Amino Acid Usage Calculator* analyses the usages of amino acid within the N1 regions. The user can select the input files to be analyzed (Figure 9A) and the results will be shown in the window (Figure 9B) or copied to the clipboard (Figure 9C). The user needs to specify a location for the output result file (Figure 9D). The analysis can be started by clicking the “*Calculate*” bar (Figure 9E). As an example, the results of amino acids usage in the N1 region of the Z417 test sequences are shown in Excel format (Figure 9F). Such results can be easily converted to different type of displays for presentation or publication. For example, the amino acid usage is presented in a bar graph in Figure 9G.

### The VDJ frequency calculator

**Figure 10:**
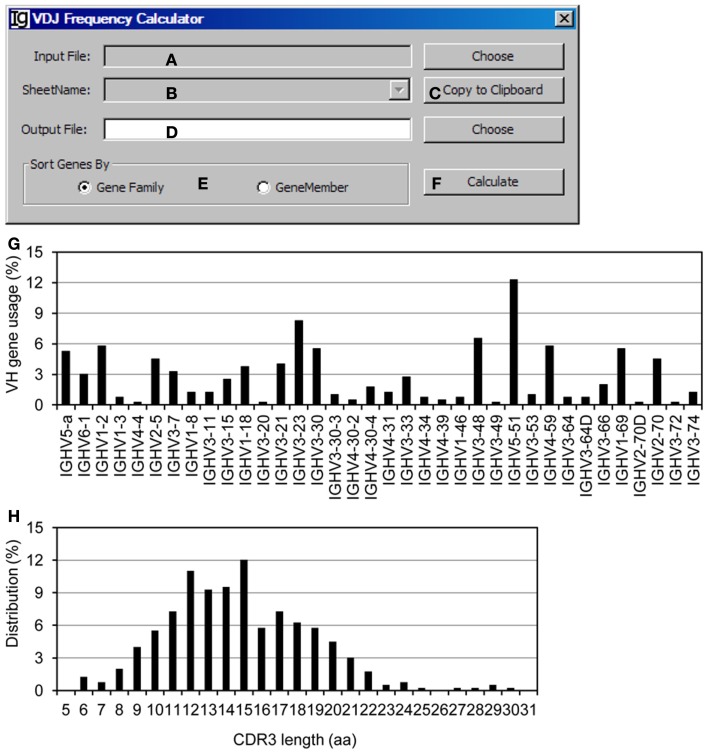
**The VDJ frequency calculator**. Diagram shows the interface of *the VDJ Frequency Calculator*. **(A)** Button to select the input footprint analysis result file. **(B)** Combo box for selecting the sheet for processing, when an Excel file is selected as the input file. **(C)** Button to copy the value in **(B)** to clipboard. **(D)** Button to choose the output file. **(E)** Radio button group to select the sorting criterion for the output results. **(F)** Button to start the calculator. **(G)** The output results of V_H_ gene usage in the test sequences were presented as a bar graph. **(H)** Distribution of the Z417 test IgH gene sequences with different CDR3 lengths.

The *VDJ Frequency Calculator* calculates the frequencies of V, D, J gene usages and IgH gene CDR3 length. *Input Files* can be selected (Figure 10A) from V_H_ replacement footprint analysis result file in either CSV format or Excel format, as output by the *Footprint Analyzer* or the *Keyword Group Picker*, respectively. If the input files are in Excel format, it will populate the combo box with names of sheets containing the V_H_ replacement footprint analysis results (Figure 10B) or copied to the clipboard (Figure 10C). The user needs to specify the location of the output result file (Figure 10D). The output results can be ranked according to the V_H_ gene family or the V_H_ gene name (Figure 10E). The analysis can be started by clicking the *Calculate* bar (Figure 10F). As an example, the results of the usages different V_H_ genes in the Z417 test sequences were calculated (Figure 10G); the frequencies of V_H_ replacement footprints in the N1 or N2 regions of IgH genes using each V_H_ germline gene are also listed in the output file (not shown); and the distribution of IgH genes with different CDR3 length was also calculated (Figure 10H).

### The clonal stripper

**Figure 11:**
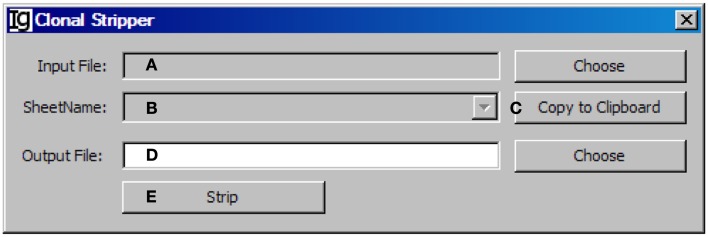
**The clonal stripper**. Diagram shows the interface of the Clonal Stripper. **(A)** Button to choose the input footprint analysis result file, which can be CSV file generated by the footprint analyzer or Excel file generated by the Keyword Group Picker. **(B)** Combo box for selecting the sheet for analysis, if an Excel file is selected in **(A)**. **(C)** Button to copy the name of selected sheet to the clipboard. **(D)** Button to choose the output file. **(E)** Button to start the stripping process.

To focus on analysis of the unique IgH sequences in any dataset, we designed the *Clonal Stripper* functional module. The *Clonal Stripper* removes redundant sequences based on their identical CDR3 regions. Input files can be selected from the results of either the Footprint Analyzer or the Keyword Group Picker, in CSV or Excel format, respectively (Figure 11A). The name of the analyzed result files will be shown in the window (Figure 11B) or copied to the clipboard (Figure 11C). The user needs to specify a location for the output result file (Figure 11D). After stripping (Figure 11E), the results will be saved as a CSV file in the same format as the output result by the *Footprint Analyzer*. Within the Z417 test sequences, there are three repeated sequences, which can be identified and eliminated by the clonal striper function (data not shown).

### The GenBank file tailor

**Figure 12:**
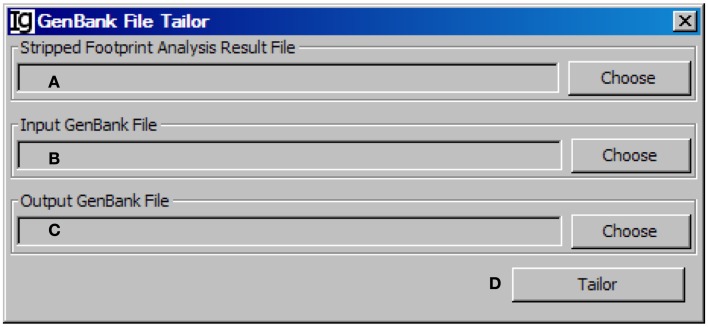
**The GenBank file tailor**. Diagram shows the interface of the *GenBank File Tailor*. **(A)** Button to choose the footprint analysis result file. **(B)** Button to choose the input GenBank file for tailoring. **(C)** Button to choose the output file. **(D)** Button to start the tailoring process.

After stripping off IgH sequences with identical CDR3 regions, the *GenBank File Tailor* function module reanalyze the GenBank files according to stripped sequence files to get rid of the repeated sequences from the GenBank record IDs (Figure 12) and save the rest unique sequences into a new FASTA file.

### The mutation analyzer

**Figure 13:**
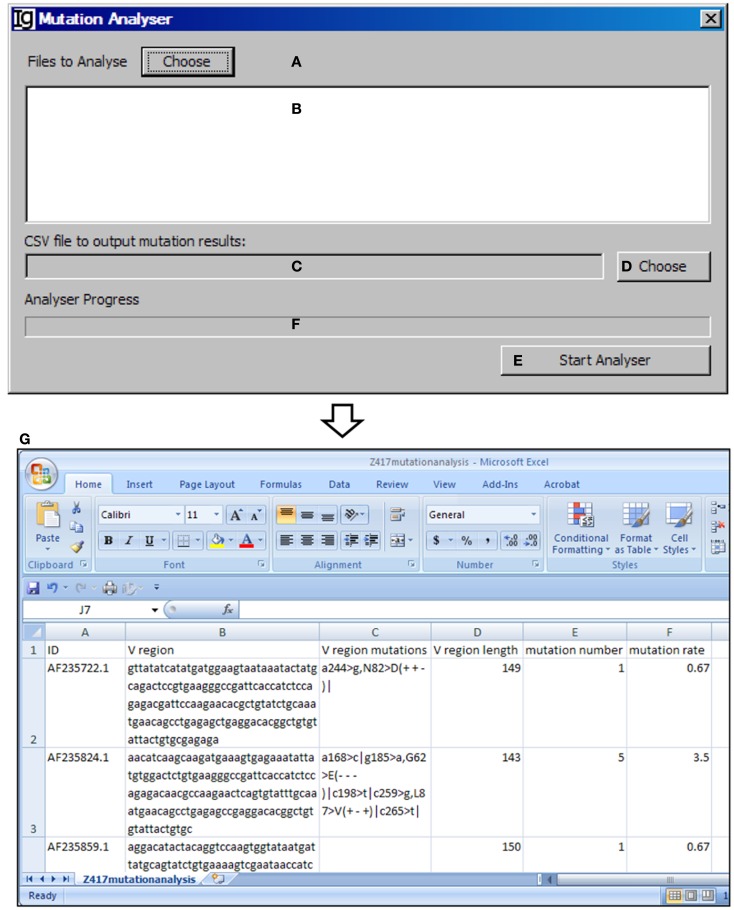
**The mutation analyzer**. Diagram shows the interface of the *Mutation Analyzer*. **(A)** Button to choose the Excel file as downloaded from IMGT/V-QUEST. **(B)** Window area for displaying selected Excel files. **(C)** Textbox for displaying path of output mutation result file. **(D)** Button for selecting output file displayed in **(C)**. **(E)** Button to the start the analyzer. **(F)** Progress bar for showing the progress of analysis. **(G)** The mutation analysis results of the Z417 test sequences. Results show the sequence ID, V region, location of each mutation within V region, V region length, mutation number, and mutation rate.

The *Mutation Analyzer* uses the results retrieved from the IMGT/V-QUEST program by the *IMGT Downloader* to calculate the number of mutations within the V_H_ region and mutation rate (Figures 13A–D). The analysis can be started by clicking the “*Start Analyser*” bar (Figure 13E), and the progress will be indicated in the window in Figure 13F. As an example of the output results, the position of the mutation within the V_H_ gene, the length of the V_H_ gene, the mutation number, and the mutation rate of each IgH gene are listed in the Excel file (Figure 13G).

### The mutation matcher

**Figure 14:**
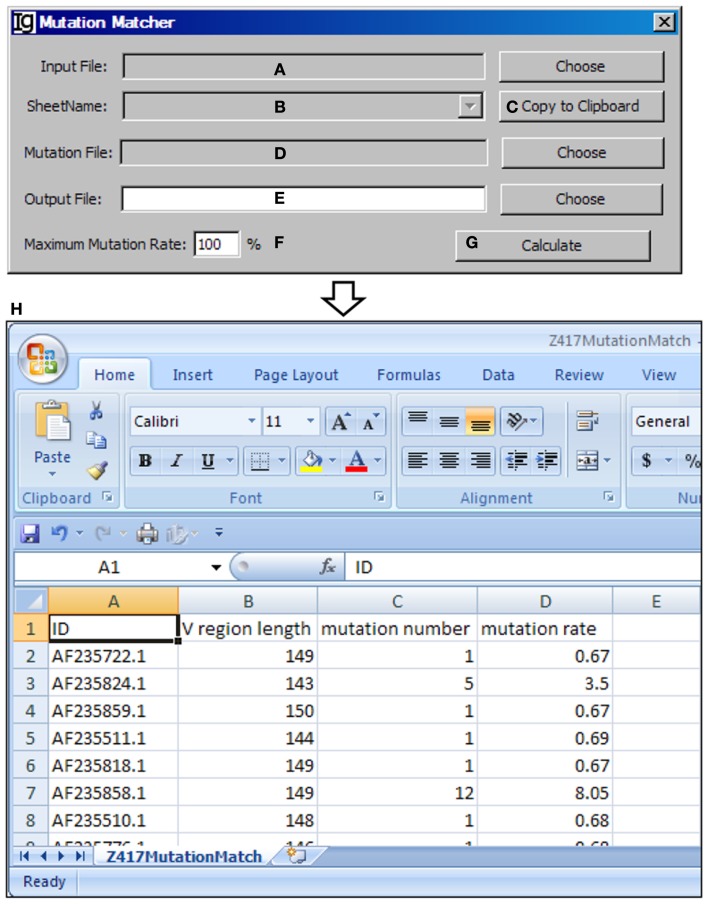
**The mutation matcher**. Diagram shows the interface of the *Mutation Matcher*. **(A)** Button for choosing the footprint analysis result file. **(B)** Combo box for selecting a sheet if a Excel file is selected. **(C)** Button to copy the name of selected sheet to the clipboard. **(D)** Button to choose the mutation analysis result file from the Mutation Analyzer. **(E)** Button to choose the output file. **(F)** Textbox to set the maximum allowed mutation rate in the V_H_ region. **(G)** Button to start the matching process. **(H)** The result file of the Z417 test sequences in Excel format.

The *Mutation Matcher* recalculates the mutation analysis results of a subgroup of V_H_ replacement analysis results according to the results obtained from the *Mutation Analyzer*. Input file can be selected from the result files from the *Footprint Analyzer* or the *Keyword Group Picker* (Figure 14A). For the latter, names of sheets containing footprint analysis results will populate the combo box (Figure 14B) or copied to the clipboard (Figure 14C). The mutation file should contain the mutation results for all the sequences (Figure 14D). The user needs to specify a location for the output result file (Figure 14E) and a maximum mutation rate (Figure 14F). Analysis can be started by clicking the *Calculate* bar (Figure 14G). An example of the output result is shown in the Excel format (Figure 14H).

### The footprint result splitter

**Figure 15:**
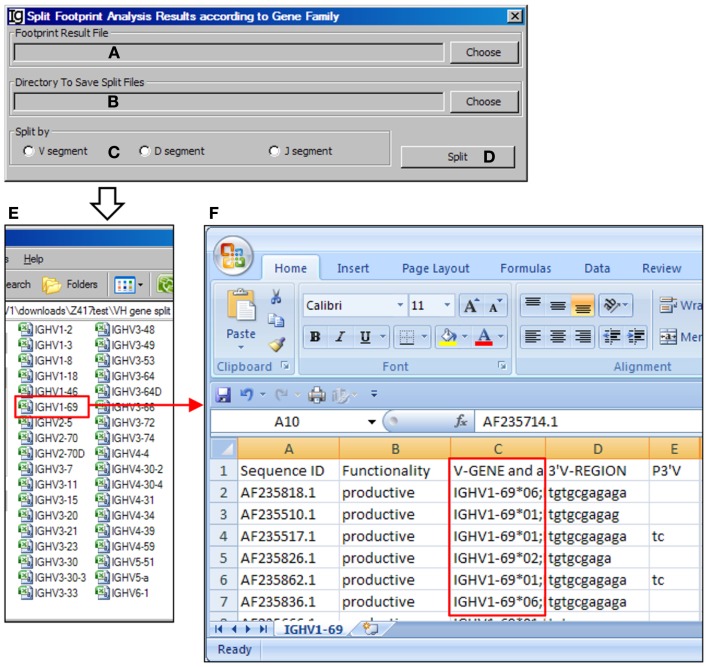
**The footprint result splitter**. Diagram shows the interface of the *Footprint Result Splitter*. **(A)** Button to select the footprint analysis result file. **(B)** Button to select the output directory. **(C)** Radio button group to select the criterion for the splitting results, according to the V, D, or J gene family. **(D)** Button to start the splitting process. **(E)** The split results according to individual V_H_ germline gene are deposited at a user specified location. **(F)** The example of V_H_ replacement footprint analysis results of IgH genes using the V_H1–69_ gene (highlighted in *red box*).

The *Footprint Result Splitter* reanalyzes the footprint analysis results according to their V_H_, D_H_, or J_H_ genes. The input files (Figure 15A) should be in CSV format, as generated by the *Footprint Analyzer*. The user needs to specify the location of the output result files (Figure 15B). The results can be split based on the V_H_ genes, D_H_ genes, or the J_H_ genes (Figure 15C) and the analysis can be started by clicking the *Split* bar (Figure 15D). The results will be saved as individual files for each germline V_H_ gene in user specified location, as shown in Figure 15E. For example, the IGHV1–69 file contains the results of all the IgH genes using the V_H1–69_ germline gene (Figure 15F).

## Discussion

In summary, we have developed a Java-based computer program, V_H_RFA-I, to analyze large number of IgH gene sequences from human or mouse origin and to identify and analyze potential V_H_ replacement products. The different functions of the V_H_RFA-I program are described in this report along with the results at each step of analysis using the Z417 test sequences. This program will be especially useful to explore the biological significance of V_H_ replacement products in human and mouse. Currently, there is no such program available.

We have included multiple functional modules in this program to analyze the frequencies of V_H_ replacement products according to their publication, keywords, V_H_, D_H_, J_H_ gene usages, and mutation status. Using such functions, we can determine the distribution of V_H_ replacement products in IgH genes derived from different diseased subjects. The V_H_RFA-I program can also identify the amino acids contributed by the potential V_H_ replacement footprints and calculated the usages of different amino acids. The V_H_RFA-I program can correlate the mutation status of the identified potential V_H_ replacement products, which will provide information regarding the selection of such V_H_ replacement products during immune response. Another advantage of the V_H_RFA-I program is that it can quickly identify potential V_H_ replacement footprints at different lengths, such as 3-, 4-, 5-, 6-, and 7-mer. Such analysis cannot be done without computer help. Clearly, with shorter length of footprint motifs, there are higher frequencies of V_H_ replacement products. Unfortunately, there is no experimental approach to determine whether the 3-, 4-, or 5-mer of V_H_ replacement footprints are more representative of the true occurrence of V_H_ replacement. For all the data analyses, we arbitrarily chose 5-mer footprint motifs to calculate the frequencies of V_H_ replacement products. Using the V_H_RFA-1 program, we have finished analyses of the 17,000 murine IgH gene sequences (32) and the 60,000 human IgH gene sequences available from the NCBI database (results will be published in separate studies). The results obtained in these studies revealed a significant contribution of V_H_ replacement products to the antibody repertoires in human and mice.

Like any other sequence analysis based method, the V_H_RFA-1 program also has its limitations. The V_H_RFA-1 program can search for the existence of V_H_ replacement footprints purely based on sequence analysis. It can identify V_H_ replacement footprints in the N1 regions as well as the N2 regions. Clearly, V_H_ replacement can only contribute footprints to the N1 regions. The identified “footprints” in the N2 regions can only be generated by random nucleotide addition. Statistical analysis results indicated that the frequencies of V_H_ replacement footprints with different lengths in the N1 regions are significantly higher than that in the N2 regions (32), which supports the sequence analysis based method to the identification of potential V_H_ replacement products. The V_H_RFA-1 program relies on the IMGT/V-Quest online service to assign the potential V_H_, D_H_, and J_H_ gene usage, which is a critique step for subsequent identification of V_H_ replacement footprints in the V_H_–D_H_ junction. In certain IgH sequence analysis, we do notice that the IMGT V_H_, D_H_, or J_H_ gene assignment might not be correct, which leads to the mistake in the identification of potential V_H_ replacement footprints. Another issue that also affects the identification of V_H_ replacement footprints is the potential existence of multiple D_H_ gene segments within IgH genes. Although it is still under debate, the latest version of the IMGT/V-Quest program has already included the option to assign up to three potential D_H_ gene segments within the V_H_ to J_H_ regions based on the standard stringency. Surprisingly, there are many IgH genes that contain multiple potential D_H_ gene segments (explored in separate studies). The existence of multiple D_H_ gene segments will change the assignment of the N1 and N2 regions and thus affect the identification of V_H_ replacement footprints. The current version of the V_H_RFA-1 program only works with the default setting in the IMGT/V-Quest program, which identifies one D_H_ gene segment for each IgH genes. The multiple D_H_ gene segments assignment results have a different output format, which is not suitable for the V_H_RFA-I program.

In our previous studies, we considered both the 5-mer V_H_ replacement footprint (5-0 method) and the 6-mer V_H_ replacement footprint with one nucleotide mismatch (6-1 method) to identify potential V_H_ replacement products (27, 37). The current version of the V_H_RFA-1 program only use the non-mutated potential V_H_ replacement footprint motif library derived from V_H_ germline genes. In this setting, mutated V_H_ replacement footprint motif within the V_H_–D_H_ junction cannot be identified by the current program. We are still developing the next version of computer program to tolerate one nucleotide mismatch within a 6-mer of V_H_ replacement footprint motif.

In summary, the V_H_RFA-I program offers a computational tool to analyze large numbers of IgH gene sequences to identify and analyze potential V_H_ replacement products in human and mice.

## Conflict of Interest Statement

The authors declare that the research was conducted in the absence of any commercial or financial relationships that could be construed as a potential conflict of interest.

## Supplementary Material

The Supplementary Material for this article can be found online at http://www.frontiersin.org/Journal/10.3389/fimmu.2014.00040/abstract

## Footnotes

^1^http://opencsv.sourceforge.net/

^2^http://jexcelapi.sourceforge.net/

^3^http://www.imgt.org/IMGT_vquest/vquest
