# Supplementary material for: VH Replacement Footprint Analyzer-I, a Java-Based Computer Program for Analyses of Immunoglobulin Heavy Chain Genes and Potential VH Replacement Products in Human and Mouse
Source: Front Immunol. 2014 Feb 10;5:40. doi: 10.3389/fimmu.2014.00040 (PMC3918983; doi:10.3389/fimmu.2014.00040)
Supplement: Supplementary file 1 [file 70319_Zhang_Presentation1.PDF]

Supplemental Table 1a. Potential Human V_H Genes that can be Targeted for V_H Replacement Recombination

Gene	3' to cRSS	3' to without cRSS	Functionality
IGHV1/OR15-1*01	TACTGTGCGAGA	CGAGA	noFuncSign
IGHV1/OR15-1*02	TACTGTGCGAGAGA	CGAGAGA	noFuncSign
IGHV1/OR15-1*03	TACTGTGCGAGAGA	CGAGAGA	noFuncSign
IGHV1/OR15-1*04	TACTGTGCGAGAGA	CGAGAGA	noFuncSign
IGHV1/OR15-2*01	TACTGTGCGAGAGA	CGAGAGA	noFuncSign
IGHV1/OR15-2*02	TACTGTGCGAGAGA	CGAGAGA	noFuncSign
IGHV1/OR15-2*03	TACTGTGCGAGAGA	CGAGAGA	noFuncSign
IGHV1/OR15-3*01	TACTGTGCGAGA	CGAGA	noFuncSign
IGHV1/OR15-3*02	TACTGTGCGAGAGA	CGAGAGA	noFuncSign
IGHV1/OR15-3*03	TACTGTGCGAGA	CGAGA	noFuncSign
IGHV1/OR15-4*01	TACTGTGCGAGA	CGAGA	noFuncSign
IGHV1/OR15-5*01	TACTGTGTGAGA	TGAGA	noFuncSign
IGHV1/OR15-5*02	TACTGTGTGAGA	TGAGA	noFuncSign
IGHV1/OR15-9*01	TACTGTGTGAGAGA	TGAGAGA	noFuncSign
IGHV1/OR21-1*01	TACTGTGTGAGAGA	TGAGAGA	noFuncSign
IGHV1-18*03	TACTGTGCGAGAGA	CGAGAGA	F
IGHV1-2*01	TACTGTGCGAGAGA	CGAGAGA	F
IGHV1-2*02	TACTGTGCGAGAGA	CGAGAGA	F
IGHV1-2*03	TACTGTGCGAGAGA	CGAGAGA	F
IGHV1-2*04	TACTGTGCGAGA	CGAGA	F
IGHV1-2*05	TACTGTGCGAGAGA	CGAGAGA	F
IGHV1-24*01	TACTGTGCAACAGA	CAACAGA	F
IGHV1-3*01	TACTGTGCGAGAGA	CGAGAGA	F
IGHV1-3*02	TACTGTGCGAGAGA	CGAGAGA	F
IGHV1-18*01	TACTGTGCGAGAGA	CGAGAGA	F
IGHV1-18*02			(F)
IGHV1-45*01	TACTGTGCAAGANA	CAAGANA	F
IGHV1-45*02	TACTGTGCAAGATA	CAAGATA	F
IGHV1-45*03	TACTGTGCAAGA	CAAGA	F
IGHV1-46*01	TACTGTGCGAGAGA	CGAGAGA	F
IGHV1-46*02	TACTGTGCGAGAGA	CGAGAGA	F
IGHV1-46*03	TACTGTGCTAGAGA	CTAGAGA	F
IGHV1-58*01	TACTGTGCGGCAGA	CGGCAGA	F
IGHV1-58*02	TACTGTGCGGCAGA	CGGCAGA	F
IGHV1-68*01	TACTGGGCAAGATA		P
IGHV1-69*01	TACTGTGCGAGAGA	CGAGAGA	F
IGHV1-69*02	TACTGTGCGAGA	CGAGA	F
IGHV1-69*03			F
IGHV1-69*04	TACTGTGCGAGAGA	CGAGAGA	F
IGHV1-69*05	TACTGTGCGAGA	CGAGA	F
IGHV1-69*06	TACTGTGCGAGAGA	CGAGAGA	F
IGHV1-69*07			F
IGHV1-69*08	TACTGTGCGAGAGA	CGAGAGA	F
IGHV1-69*09	TACTGTGCGAGAGA	CGAGAGA	F
IGHV1-69*10	TACTGTGCGAGAGA	CGAGAGA	F
IGHV1-69*11	TACTGTGCGAGAGA	CGAGAGA	F
IGHV1-69*12	TACTGTGCGAGAGA	CGAGAGA	F
IGHV1-69*13	TACTGTGCGAGAGA	CGAGAGA	(F)
IGHV1-8*01	TACTGTGCGAGAGG	CGAGAGG	F
IGHV1-8*02	TACTGTGCGAGAGG	CGAGAGG	F
IGHV1-c*01	TACTATGCAAGA		ORF
IGHV1-f*01	TACTGTGCAACA	CAACA	F
IGHV1-f*02			F
IGHV1-NL1*01	TACTGTGTGAGAGA	TGAGAGA	P

IGHV2/OR16-5*01	TACTGTGCATGGA	CATGGA	noFuncSign
IGHV2-5*01	TACTGTGCACACAGACC	CACACAGACC	F
IGHV2-5*02			F
IGHV2-5*03			F
IGHV2-5*04	TACTGTGTACGG	TACGG	F
IGHV2-5*05	TACTGTGCACACAGAC	CACACAGAC	F
IGHV2-5*06	TACTGTGCACACAGA	CACACAGA	F
IGHV2-5*07	TACTGTGTA	TA	F
IGHV2-5*08	TACTGTGCACACAGAC	CACACAGAC	F
IGHV2-5*09	TACTGTGCACACAGAC	CACACAGAC	F
IGHV2-5*10	TACTGTGCACGG	CACGG	F
IGHV2-10*01	TACTGTGCAAGGAGAC	CAAGGAGAC	P
IGHV2-26*01	TACTGTGCACGGATAC	CACGGATAC	F
IGHV2-70*01	TACTGTGCACGGATAC	CACGGATAC	F
IGHV2-70*02	TACTG		F
IGHV2-70*03	TACTG		F
IGHV2-70*04	TAC		F
IGHV2-70*05			F
IGHV2-70*06	TACTG		F
IGHV2-70*07	TACTG		F
IGHV2-70*08	TACTG		F
IGHV2-70*09	TACTGTGTACGG	TACGG	F
IGHV2-70*10	TACTGTGCACGGATAC	CACGGATAC	F
IGHV2-70*11	TACTGTGCACGGATAC	CACGGATAC	F
IGHV2-70*12	TACTGTGCACACAGAC	CACACAGAC	F
IGHV2-70*13	TATTGTGCACGGATAC		F
IGHV3/OR15-7*01	TACTGTGCTAGA	CTAGA	noFuncSign
IGHV3/OR15-7*02	TACTGTGCTAGA	CTAGA	noFuncSign
IGHV3/OR15-7*03	TACTGTGCTAGA	CTAGA	noFuncSign
IGHV3/OR15-7*05	TACTGTGCTAGAGA	CTAGAGA	noFuncSign
IGHV3/OR16-10*01	TACTGTGCAAGA	CAAGA	noFuncSign
IGHV3/OR16-10*02	TACTGTGCAAGA	CAAGA	noFuncSign
IGHV3/OR16-10*03	TACTGTGCAAGAGA	CAAGAGA	noFuncSign
IGHV3/OR16-12*01	TACTGTGCAAGA	CAAGA	noFuncSign
IGHV3/OR16-13*01	TACTGTACTAGA		noFuncSign
IGHV3/OR16-14*01	TACTGTACTAGA		noFuncSign
IGHV3/OR16-15*01	TACTGTGTGAGAAA	TGAGAAA	noFuncSign
IGHV3/OR16-15*02	TACTGTGTGAGA	TGAGA	noFuncSign
IGHV3/OR16-16*01	TACTGTGTGAGA	TGAGA	noFuncSign
IGHV3/OR16-6*02	TACTGTACCACAGG		noFuncSign
IGHV3/OR16-8*01	TACTGTGTGA	TGA	noFuncSign
IGHV3/OR16-8*02	TACTGTGTGAAACA	TGAAACA	noFuncSign
IGHV3/OR16-9*01	TACTGTGTGA	TGA	noFuncSign
IGHV3-9*01	TACTGTGCAAAAGATA	CAAAAGATA	F
IGHV3-9*02	TACTGTGCAAAAGATA	CAAAAGATA	F
IGHV3-11*01	TACTGTGCGAGAGA	CGAGAGA	F
IGHV3-11*03	TACTGTGCGAGA	CGAGA	F
IGHV3-11*04	TACTGTGCGAGAGA	CGAGAGA	F
IGHV3-13*01	TACTGTGCAAGAGA	CAAGAGA	F
IGHV3-13*02	TACTGTGCAAGAGA	CAAGAGA	F
IGHV3-13*03	TACTGTGCAAGA	CAAGA	F
IGHV3-13*04	TACTGTGCAAGAGA	CAAGAGA	F
IGHV3-15*01	TACTGTACCACAGA		F
IGHV3-15*02	TACTGTACCACAGA		F
IGHV3-15*03	TACTGTACCACAGA		F
IGHV3-15*04	TACTGTACCACAGA		F
IGHV3-15*05	TACTGTACCACAGA		F

IGHV3-15*06	TACTGTACCACAGA		F
IGHV3-15*07	TACTGTACCACAGA		F
IGHV3-15*08	TACTGTACCACAGG		F
IGHV3-16*01	TACTGTGTGAGAAA	TGAGAAA	ORF
IGHV3-16*02	TACTGTGTGAGAAA	TGAGAAA	ORF
IGHV3-19*01	TACTGTGTGAGAAA	TGAGAAA	P
IGHV3-20*01	CACTGTGCGAGAGA		F
IGHV3-21*01	TACTGTGCGAGAGA	CGAGAGA	F
IGHV3-21*02	TACTGTGCGAGAGA	CGAGAGA	F
IGHV3-21*03	TACTGTGCGAGAGA	CGAGAGA	F
IGHV3-21*04	TACTGTGCGAGAGA	CGAGAGA	F
IGHV3-22*01	TACTGTTCCAGAGA		P
IGHV3-22*02	TACTGTTCCAGAGA		P
IGHV3-23*01	TACTGTGCGAAAGA	CGAAAGA	F
IGHV3-23*02	TACTGTGCGAAAGA	CGAAAGA	F
IGHV3-23*03	TACTGTGCGAAA	CGAAA	F
IGHV3-23*04	TACTGTGCGAAAGA	CGAAAGA	F
IGHV3-23*05	TACTGTGCGAAA	CGAAA	F
IGHV3-25*01	TAGTGTACCAGAGA		P
IGHV3-25*02	TAGTGTACCAGAGA		P
IGHV3-25*03	TAGTGTACCAGA		P
IGHV3-25*04	TACTGTACCAGA		ORF
IGHV3-25*05	TAGTGTACCAGAGA		P
IGHV3-30*01	TACTGTGCGAGAGA	CGAGAGA	F
IGHV3-30*02	TACTGTGCGAAAGA	CGAAAGA	F
IGHV3-30*03	TACTGTGCGAGAGA	CGAGAGA	F
IGHV3-30*04	TACTGTGCGAGAGA	CGAGAGA	F
IGHV3-30*05	TACTGTGCGAGAGA	CGAGAGA	F
IGHV3-30*06	TACTGTGCGAGAGA	CGAGAGA	F
IGHV3-30*07	TACTGTGCGAGAGA	CGAGAGA	F
IGHV3-30*08	TACTGTGCGAGA	CGAGA	F
IGHV3-30*09	TACTGTGCGAGAGA	CGAGAGA	F
IGHV3-30*10	TACTGTGCGAGAGA	CGAGAGA	F
IGHV3-30*11	TACTGTGCGAGAGA	CGAGAGA	F
IGHV3-30*12	TACTGTGCGAGAGA	CGAGAGA	F
IGHV3-30*13	TACTGTGCGAGAGA	CGAGAGA	F
IGHV3-30*14	TACTGTGCGAGAGA	CGAGAGA	F
IGHV3-30*15	TACTGTGCGAGAGA	CGAGAGA	F
IGHV3-30*16	TACTGTGCGAGAGA	CGAGAGA	F
IGHV3-30*17	TACTGTGCGAGAGA	CGAGAGA	F
IGHV3-30*18	TACTGTGCGAAAGA	CGAAAGA	F
IGHV3-30*19	TACTGTGCGAGAGA	CGAGAGA	F
IGHV3-30-3*01	TACTGTGCGAGA	CGAGA	F
IGHV3-30-3*02	TACTGTGCGAAAGA	CGAAAGA	F
IGHV3-32*01	GGCTATACATAAGGTC		P
IGHV3-33*01	TACTGTGCGAGAGA	CGAGAGA	F
IGHV3-33*02	TACTGTGCGAGAGA	CGAGAGA	F
IGHV3-33*03	TACTGTGCGAAAGA	CGAAAGA	F
IGHV3-33*04	TACTGTGCGAGAGA	CGAGAGA	F
IGHV3-33*05	TACTGTGCGAGAGA	CGAGAGA	F
IGHV3-33*06	TACTGTGCGAAAGA	CGAAAGA	F
IGHV3-35*01	TACTGTGTGAGAAA	TGAGAAA	ORF
IGHV3-38*01	TACTGTGCCAGATATA	CCAGATATA	ORF
IGHV3-38*02	TACTGTGCCAGATATA	CCAGATATA	ORF
IGHV3-43*01	TACTGTGCAAAAGATA	CAAAAGATA	F
IGHV3-43*02	TACTGTGCAAAA	CAAAA	F
IGHV3-47*01	TATTGTGCAAGA		P

IGHV3-47*02	TATTGTGCAAGAGA		P
IGHV3-47*03	TATG		P
IGHV3-48*01	TACTGTGCGAGAGA	CGAGAGA	F
IGHV3-48*02	TACTGTGCGAGAGA	CGAGAGA	F
IGHV3-48*03	TACTGTGCGAGAGA	CGAGAGA	F
IGHV3-48*04	TACTGTGCGAGAGA	CGAGAGA	F
IGHV3-49*01	TACTGTACTAGAGA		F
IGHV3-49*02	TACTGTACTAGAGA		F
IGHV3-49*03	TACTGTACTAGAGA		F
IGHV3-49*04	TACTGTACTAGAGA		F
IGHV3-49*05	TACTGTACTAGAGA		F
IGHV3-52*01	TACTGTGTGAGAGG	TGAGAGG	P
IGHV3-52*02	TACTGTGTGAGA	TGAGA	P
IGHV3-52*03	TACTGTGTGAGA	TGAGA	P
IGHV3-53*01	TACTGTGCGAGAGA	CGAGAGA	F
IGHV3-53*02	TACTGTGCGAGA	CGAGA	F
IGHV3-53*03	TACTGTGCTAGGGA	CTAGGGA	F
IGHV3-53*04	TACTGTGCGAGAGA	CGAGAGA	F
IGHV3-54*01	TACTGTATGTGAGY		P
IGHV3-54*02	TACTGTATGTGAGG		P
IGHV3-54*04	TACTGTATGTGAGT		P
IGHV3-62*01	TACTGTGTGAAAGA	TGAAAGA	P
IGHV3-63*01	GGCTGTACATAAGGTT		P
IGHV3-63*02	GGCTGTACATAA		P
IGHV3-64*01	TACTGTGCGAGAGA	CGAGAGA	F
IGHV3-64*02	TACTGTGCGAGAGA	CGAGAGA	F
IGHV3-64*03	TACTGTGTGAAAGA	TGAAAGA	F
IGHV3-64*04	TACTGTGCGAGAGA	CGAGAGA	F
IGHV3-64*05	TACTGTGTGAAAGA	TGAAAGA	F
IGHV3-66*01	TACTGTGCGAGAGA	CGAGAGA	F
IGHV3-66*02	TACTGTGCGAGA	CGAGA	F
IGHV3-66*03	TACTGTGCGAGAGA	CGAGAGA	F
IGHV3-66*04	TACTGTGCGAGACA	CGAGACA	F
IGHV3-7*01	TACTGTGCGAGAGA	CGAGAGA	F
IGHV3-7*02	TACTGTGCGAGA	CGAGA	F
IGHV3-7*03	TACTGTGCGAGAGA	CGAGAGA	F
IGHV3-71*01	TACTGTGCGAGAGA	CGAGAGA	P
IGHV3-71*02	TACTGTGCGAGAGA	CGAGAGA	P
IGHV3-71*03	TACTGTGCGAGAGA	CGAGAGA	P
IGHV3-72*01	TACTGTGCTAGAGA	CTAGAGA	F
IGHV3-72*02			F
IGHV3-73*01	TACTGTACTAGACA		F
IGHV3-73*02	TACTGTACTAGACA		F
IGHV3-74*01	TACTGTGCAAGAGA	CAAGAGA	F
IGHV3-74*02	TACTGTGCAAGA	CAAGA	F
IGHV3-74*03	TACTGTGCAAGAGA	CAAGAGA	F
IGHV3-d*01	TACTGTAAGAAA		F
IGHV3-h*01	TACTGTGCGAGAGA	CGAGAGA	P
IGHV3-h*02	TACTGTGCGAGAGA	CGAGAGA	P
IGHV3-NL1*01	TACTGTGCGAAAGA	CGAAAGA	F
IGHV4/OR15-8*01	TACTGTGCGAGAGA	CGAGAGA	noFuncSign
IGHV4/OR15-8*02	TACTGTGCGAGAGA	CGAGAGA	noFuncSign
IGHV4/OR15-8*03	TACTGTGCGAGAGA	CGAGAGA	noFuncSign
IGHV4-4*01	TGCTGTGCGAGAGA		F
IGHV4-4*02	TACTGTGCGAGAGA	CGAGAGA	F
IGHV4-4*03	TACTG		F
IGHV4-4*04	TACTG		F

IGHV4-4*05	TACTG		F
IGHV4-4*06	TACTGTGCGAGAGA	CGAGAGA	F
IGHV4-4*07	TACTGTGCGAGAGA	CGAGAGA	F
IGHV4-28*01	TACTGTGCGAGAAA	CGAGAAA	F
IGHV4-28*02	TACTGTGCGAGAAA	CGAGAAA	F
IGHV4-28*03	TACTGTGCGAGAGA	CGAGAGA	F
IGHV4-28*04	TACTGTGCGAGA	CGAGA	F
IGHV4-28*05	TACTG		F
IGHV4-28*06	TACTGTGCGAGAAA	CGAGAAA	F
IGHV4-30-2*01	TACTGTGCCAGAGA	CCAGAGA	F
IGHV4-30-2*02	TACTGTGCG	CG	F
IGHV4-30-2*03	TACTGTGCGAGACA	CGAGACA	F
IGHV4-30-2*04	TACTGTGCGAGAGA	CGAGAGA	F
IGHV4-30-2*05	TACTGTGCCAGAGA	CCAGAGA	F
IGHV4-30-4*01	TACTGTGCCAGAGA	CCAGAGA	F
IGHV4-30-4*02	TACTGTGCCAGAGA	CCAGAGA	F
IGHV4-30-4*03	TACTG		F
IGHV4-30-4*04	TACTG		F
IGHV4-30-4*05	TACTGTGCCAGAGA	CCAGAGA	F
IGHV4-30-4*06	TACTGTGCCAGAGA	CCAGAGA	F
IGHV4-31*01	TACTGTGCGAGAGA	CGAGAGA	F
IGHV4-31*02	TACTGTGCGAGAGA	CGAGAGA	F
IGHV4-31*03	TACTGTGCGAGAGA	CGAGAGA	F
IGHV4-31*04	TACTGTGCG	CG	F
IGHV4-31*05	TACTGTGCG	CG	F
IGHV4-31*06	TACTG		F
IGHV4-31*07	TACTG		F
IGHV4-31*08	TACTG		F
IGHV4-31*09	TACTG		F
IGHV4-31*10	TACTGTGCGAGAGA	CGAGAGA	F
IGHV4-34*01	TACTGTGCGAGAGG	CGAGAGG	F
IGHV4-34*02	TACTGTGCGAGAGG	CGAGAGG	F
IGHV4-34*03	TACTG		F
IGHV4-34*04	TACTGTGCGAGAGG	CGAGAGG	F
IGHV4-34*05	TACTGTGCGAGAGG	CGAGAGG	F
IGHV4-34*06	TACTG		F
IGHV4-34*07	TACTG		F
IGHV4-34*08	TACTGTGCG	CG	F
IGHV4-34*09	TACTGTGCGAGAGA	CGAGAGA	F
IGHV4-34*10	TACTGTGCGAGATA	CGAGATA	F
IGHV4-34*11	TGCTGTGCGAGAGA		F
IGHV4-34*12	TACTGTGCGAGA	CGAGA	F
IGHV4-34*13	TACTGTGCGAGAGG	CGAGAGG	F
IGHV4-39*01	TACTGTGCGAGACA	CGAGACA	F
IGHV4-39*02	TACTGTGCGAGAGA	CGAGAGA	F
IGHV4-39*03	TACTG		F
IGHV4-39*04			F
IGHV4-39*05	TACTGTGCG	CG	F
IGHV4-39*06	TACTGTGCGAGAGA	CGAGAGA	F
IGHV4-39*07	TACTGTGCGAGAGA	CGAGAGA	F
IGHV4-55*01	TACTGTGCGAGATA	CGAGATA	P
IGHV4-55*02	TACTGTGCGAGATA	CGAGATA	P
IGHV4-55*03	TACTG		P
IGHV4-55*04	TACTG		P
IGHV4-55*05	TACTG		P
IGHV4-55*06	TACTG		P
IGHV4-55*07	TACT		P

IGHV4-55*08	TACTGTGCGAGAGA	CGAGAGA	P
IGHV4-55*09	TACTGTGCGAGAAA	CGAGAAA	P
IGHV4-59*01	TACTGTGCGAGAGA	CGAGAGA	F
IGHV4-59*02	TACTGTGCGAGAGA	CGAGAGA	F
IGHV4-59*03	TACTGTGCG	CG	F
IGHV4-59*04	TACTGTGCG	CG	F
IGHV4-59*05	TACTGTGCG	CG	F
IGHV4-59*06	TACTGTGCG	CG	F
IGHV4-59*07	TACTGTGCGAGA	CGAGA	F
IGHV4-59*08	TACTGTGCGAGACA	CGAGACA	F
IGHV4-59*09	TACTGTGCGAGAGG	CGAGAGG	F
IGHV4-59*10	TACTGTGCGAGATA	CGAGATA	F
IGHV4-61*01	TACTGTGCGAGAGA	CGAGAGA	F
IGHV4-61*02	TACTGTGCGAGAGA	CGAGAGA	F
IGHV4-61*03	TACTGTGCGAGAGA	CGAGAGA	F
IGHV4-61*04	TACTG		F
IGHV4-61*05	TACTGTGCGAGA	CGAGA	F
IGHV4-61*06	TACTGTGCCAGAGA	CCAGAGA	ORF
IGHV4-61*07	TACTGTGCGAGACA	CGAGACA	F
IGHV4-61*08	TACTGTGCGAGAGA	CGAGAGA	F
IGHV4-b*01	TACTGTGCGAGA	CGAGA	F
IGHV4-b*02	TACTGTGCGAGA	CGAGA	F
IGHV5-51*01	TACTGTGCGAGACA	CGAGACA	F
IGHV5-51*02	TACTGTGCGAGACA	CGAGACA	F
IGHV5-51*03	TACTGTGCGAGA	CGAGA	F
IGHV5-51*04	TACTGTGCGAGA	CGAGA	F
IGHV5-51*05			F
IGHV5-78*01	TATTGTGTGAGA		P
IGHV5-a*01	TACTGTGCGAGA	CGAGA	F
IGHV5-a*02	TACTGTGCGAGACA	CGAGACA	F
IGHV5-a*03	TACTGTGCGAGA	CGAGA	F
IGHV5-a*04	TACTGTGCGAGA	CGAGA	F
IGHV6-1*01	TACTGTGCAAGAGA	CAAGAGA	F
IGHV6-1*02	TACTGTGCAAGAGA	CAAGAGA	F
IGHV7-4-1*01	TACTGTGCGAGA	CGAGA	F
IGHV7-4-1*02	TACTGTGCGAGAGA	CGAGAGA	F
IGHV7-4-1*03			F
IGHV7-4-1*04	TACTGTGCGAGAGA	CGAGAGA	F
IGHV7-4-1*05	TACTGTGCGAGAGA	CGAGAGA	F
IGHV7-34-1*02	TACTGTGCGAAGTA	CGAAGTA	P
IGHV7-40*03	TACTGTGCGA	CGA	P
IGHV7-81*01	TACTGTGCGAGATA	CGAGATA	ORF

Note: F, functional; P, pseudogene; ORF, open reading frame; noFuncSign, signed as non-functional.

Supplemental Table 1b. Potential Mouse V_H Genes that can be Targeted for V_H Replacement Recombination

Gene	3' to cRSS	3' to without cRSS	Functionality
IGHV1-4*01	TACTGTGCAAGA	CAAGA	F
IGHV1-4*02	TACTGTGCAAGA	CAAGA	F
IGHV1-5*01	TACTGTACAAGA		F
IGHV1-7*01	TACTGTGCAAGA	CAAGA	F
IGHV1-8*01	TACTGTACAAG		P
IGHV1-9*01	TACTGTGCAAGA	CAAGA	F
IGHV1-11*01	TACTGTGGAAGAGG	GAAGAGG	F
IGHV1-12*01	TTCTGTGCAAGA		F
IGHV1-13*01	TATTGTGAGAGA		P
IGHV1-14*01	TACTGTGCAAGA	CAAGA	F
IGHV1-15*01	TACTGTACAAGA		F
IGHV1-16*01	TACTGTGCAAGA	CAAGA	ORF
IGHV1-17-1*01	TACTGTGCAAGA	CAAGA	F
IGHV1-18*01	TACTGTGCAAGA	CAAGA	F
IGHV1-18*02			[F]
IGHV1-18*03			[F]
IGHV1-19*01	TACTGTGCAAGA	CAAGA	F
IGHV1-19-1*01	TACCGTGCAAGA		P
IGHV1-20*01	TATTGTGCAAGA		F
IGHV1-20*02	TATTGTGCAAGA		F
IGHV1-21*01	TACTGTGCAAGA	CAAGA	P
IGHV1-21-1*01	TACTCTGCAAGA		P
IGHV1-22*01	TACTGTGCAAGA	CAAGA	F
IGHV1-23*01	TACTGTACAAGA		ORF
IGHV1-24*01	TACTGTGCAAGA	CAAGA	ORF
IGHV1-25*01	TACTGTGCAAGA	CAAGA	P
IGHV1-26*01	TACTGTGCAAGA	CAAGA	F
IGHV1-27*01	TTCTGTGCAAGA		P
IGHV1-28*01	TACTTTGCAAGA		P
IGHV1-31*01	TACTGTGCAAGA	CAAGA	F
IGHV1-32*01	TACTGTGTAAGA	TAAGA	P
IGHV1-34*01	TACTGTGCAAGA	CAAGA	F
IGHV1-34*02	TACTGTGCAAGA	CAAGA	F
IGHV1-35*01	TACCGTGCAAGACT		P
IGHV1-35*02	TACCGTGCAAGA		P
IGHV1-35*03			P
IGHV1-36*01	TACTGTGCAAGA	CAAGA	F
IGHV1-37*01	TATTGTGCAAGA		F
IGHV1-39*01	TACTGTGCAAGA	CAAGA	F
IGHV1-42*01	TACTGTGCAAGA	CAAGA	F
IGHV1-42*02			[F]
IGHV1-42*03			[P]
IGHV1-43*01	TACTGTGCAAGA	CAAGA	F
IGHV1-46*01	TACTGTGCATGG	CATGG	P
IGHV1-47*01	TACTGTGCAAGG	CAAGG	F
IGHV1-48*01	TATTGTGTAAGA		P
IGHV1-49*01	TACTGTGCAAGA	CAAGA	F
IGHV1-50*01	TACTGTGCAAGA	CAAGA	F
IGHV1-51*01	TGTTGTGCTAGACAAGTG		P
IGHV1-52*01	TACTGTGCAAGA	CAAGA	F
IGHV1-53*01	TATTGTGCAAGA		F
IGHV1-53*02			[F]
IGHV1-53*03			[F]

IGHV1-53*04			[F]
IGHV1-54*01	TTCTGTGCAAGA		F
IGHV1-54*02	TTCTGTGCAAGA		F
IGHV1-54*03	TTCTGTGCAAGA		[F]
IGHV1-55*01	TACTGTGCAAGA	CAAGA	F
IGHV1-55*02			[F]
IGHV1-55*03			[F]
IGHV1-55*04			[F]
IGHV1-56*01	TTCTGTGCAAGA		F
IGHV1-56*02	TTCTGTGCAAGA		[F]
IGHV1-58*01	TTCTGTGCAAGA		F
IGHV1-58*02	TTCTGTGCAAGA		P
IGHV1-59*01	TACTGTGCAAGA	CAAGA	F
IGHV1-60*01	TACTGTGCTAGA	CTAGA	P
IGHV1-61*01	TACTGTGCAAGA	CAAGA	F
IGHV1-62-1*01	CTGTGCAAGGAA		F
IGHV1-62-2*01	TTCTGTGCAAGACACGAAGA		F
IGHV1-62-3*01	TACTGTGCAAGA	CAAGA	ORF
IGHV1-62-3*02			[F]
IGHV1-63*01	TACTGTGCAAGA	CAAGA	F
IGHV1-63*02	TACTGTGCAAGA	CAAGA	F
IGHV1-64*01	TACTGTGCAAGA	CAAGA	F
IGHV1-64*02			[F]
IGHV1-66*01	TACTGTGCAAGA	CAAGA	F
IGHV1-67*01	TACTGTGCAAGA	CAAGA	F
IGHV1-69*01	TACTGTGCAAGA	CAAGA	F
IGHV1-69*02	TACTGTGCAAGA	CAAGA	F
IGHV1-69*03			[F]
IGHV1-70*01	TTCTGTGCAAGA		P
IGHV1-71*01	TTCTGTGCAAGACACGAAGA		F
IGHV1-71*02	TTCTGTGCAAGACACGAAGA		F
IGHV1-72*01	TATTGTGCAAGA		F
IGHV1-72*02			[F]
IGHV1-72*03			[F]
IGHV1-72*04	TATTGTGCAAGA		F
IGHV1-72*05			[F]
IGHV1-74*01	TACTGTGCAATA	CAATA	F
IGHV1-74*02			[F]
IGHV1-74*03			[F]
IGHV1-74*04	TACTGTGCAATA	CAATA	F
IGHV1-75*01	TTCTGTGCAAGA		F
IGHV1-76*01	TTCTGTGCAAGA		F
IGHV1-77*01	TTCTGTGCAAGA		F
IGHV1-78*01	TTCTGTGCAAGA		F
IGHV1-79*01	TTCTGTGCAAGA		P
IGHV1-80*01	TTCTGTGCAAGA		F
IGHV1-81*01	TTCTGTGCAAGA		F
IGHV1-82*01	TTCTGTGCAAGA		F
IGHV1-83*01	TTCTGTGCAAGA		P
IGHV1-84*01	TTCTGTGCAAGA		F
IGHV1-84*02	TTCTGTGCAAGA		[F]
IGHV1-85*01	TTCTGTGCAAGA		F
IGHV1S10*01	TTCTGTGCAAGA		P
IGHV1S10*02	TTCTGTGCAAGA		P
IGHV1S100*01			[F]
IGHV1S101*01			[P]
IGHV1S101*02			[P]

IGHV1S103*01			[F]
IGHV1S107*01			[F]
IGHV1S108*01			[F]
IGHV1S11*01	TACTGTGCAAGA	CAAGA	P
IGHV1S110*01			[P]
IGHV1S111*01			[F]
IGHV1S112*01			[P]
IGHV1S112*02			[F]
IGHV1S113*01			[F]
IGHV1S113*02			[F]
IGHV1S118*01			[F]
IGHV1S12*01	TTCTGTGCAAGA		F
IGHV1S120*01			[F]
IGHV1S120*02			[F]
IGHV1S121*01			[F]
IGHV1S122*01			[F]
IGHV1S124*01			[P]
IGHV1S126*01	TACTGTGCAAGA	CAAGA	[F]
IGHV1S127*01	TACTGTACAAGA		[F]
IGHV1S130*01	TACTGTGCAAGA	CAAGA	[F]
IGHV1S132*01	TTCTGTGCAAGA		[F]
IGHV1S134*01	TTCTGTGCAAGA		[F]
IGHV1S135*01	TACTGTGCAAGA	CAAGA	[F]
IGHV1S136*01	TACTGTGCAAGA	CAAGA	[F]
IGHV1S137*01	TACTGTGCAAGA	CAAGA	[F]
IGHV1S14*01	TACTGTGCAAGA	CAAGA	F
IGHV1S15*01	TATTGTGCAATA		P
IGHV1S16*01	TACTGTACAATA		P
IGHV1S17*01	TACTGTACAAGA		P
IGHV1S18*01	TACTGTGCAAGA	CAAGA	P
IGHV1S19*01	TTCTGTGCAAGA		P
IGHV1S20*01	TTCTGTGCAAGA		F
IGHV1S20*02	TTCTGTGCAAGA		[F]
IGHV1S21*01			F
IGHV1S21*02			[F]
IGHV1S22*01	TACTGTACAAGA		F
IGHV1S26*01	TACTGTGCAAGA	CAAGA	F
IGHV1S28*01	TACTGTTCGAGA		P
IGHV1S29*01			F
IGHV1S29*02	TACTGTGCAAGA	CAAGA	F
IGHV1S30*01	TACTGTGCAAGA	CAAGA	P
IGHV1S31*01			F
IGHV1S32*01			F
IGHV1S33*01			F
IGHV1S34*01	TACTGTGCAAGA	CAAGA	F
IGHV1S35*01	TACTGTGCAAGA	CAAGA	F
IGHV1S36*01	TACTGTACAAGA		F
IGHV1S36*02	TACTGTACAAGA		[F]
IGHV1S37*01			F
IGHV1S40*01	TACTGTGCAAGA	CAAGA	F
IGHV1S41*01	TTCTGTGCAAGA		F
IGHV1S44*01			[F]
IGHV1S45*01	TACTGTGCAAGA	CAAGA	F
IGHV1S46*01	TACTGTGCAAGAT	CAAGAT	[F]
IGHV1S47*01	TTCTGTGCAAGA		ORF
IGHV1S49*01	TTCTGCGCAAGA		F
IGHV1S5*01	TATTGTGCAAGA		F

IGHV1S50*01	TTCTGTGCAAGA		F
IGHV1S51*01	TTCTGTGCAAGA		P
IGHV1S52*01	TTCTGCGCAAGA		F
IGHV1S53*01	TTCTGTAAAAGA		F
IGHV1S53*02	TTCTGTAAAAGA		F
IGHV1S53*03	TTCTGTAAAAGA		F
IGHV1S55*01	TACTGTGCAAGA	CAAGA	F
IGHV1S56*01	TTCTGTGCAAGA		F
IGHV1S61*01	TATTGTGCAAGA		F
IGHV1S65*01			[F]
IGHV1S65*02			[P]
IGHV1S65*03			[F]
IGHV1S67*01			[F]
IGHV1S67*02			[F]
IGHV1S68*01			[F]
IGHV1S68*02			[F]
IGHV1S70*01			[F]
IGHV1S72*01			[F]
IGHV1S73*01			[F]
IGHV1S74*01			[P]
IGHV1S75*01			[F]
IGHV1S75*02			[F]
IGHV1S78*01			[F]
IGHV1S81*01			[F]
IGHV1S81*02	TACTGTGCAAGA	CAAGA	[F]
IGHV1S82*01			[F]
IGHV1S83*01			[F]
IGHV1S84*01			[P]
IGHV1S87*01			[F]
IGHV1S9*01	TACTGTGCAAGA	CAAGA	P
IGHV1S92*01			[F]
IGHV1S95*01			[F]
IGHV1S96*01			[F]
IGHV10-1*01	TACTGTGTGAGACA	TGAGACA	F
IGHV10-1*02	TACTGTGTGAGCGA	TGAGCGA	F
IGHV10-3*01	TACTGTGTGAGAG	TGAGAG	F
IGHV10-3*02	TACTGTGTGAGAGA	TGAGAGA	F
IGHV10-3*03	TACTGTGTGAGAGA	TGAGAGA	F
IGHV10S3*01	TACTGTGTGAGAGA	TGAGAGA	F
IGHV10S4*01	TACTGTGTGAGA	TGAGA	F
IGHV11-1*01	TTCTGTATGAGATA		F
IGHV11-2*01	TTCTGTATGAGATA		F
IGHV11-2*02	TTCTGTATGAGATA		F
IGHV12-1*01	TACTGTTCTAGGGAAAACCA		P
IGHV12-1*02	TACTATCCCAGGGAAAACCA		P
IGHV12-1-1*01	TACTGTTCCAGGGAAAACCA		F
IGHV12-1-2*01	TACTGTTCCAGGGAAAACCA		P
IGHV12-2*01	TACTATTCCAGGGAAAACCA		P
IGHV12-2-1*01	TACTATTCCAGGGAAAACCA		ORF
IGHV12-3*01	TACTGTGCAGGAAGACAG	CAGGAAGACAG	F
IGHV12-3*02	TACTGTGCAGGAGACAGA	CAGGAGACAGA	F
IGHV13-1*01	TACTGTGCAAGAGAGA	CAAGAGAGA	F
IGHV13-1*02	TACTGGGCAAGAGAGA		ORF
IGHV13-2*01	TTTTGTAGTAGA		F
IGHV13-2*02	TATTGTAGTAGA		F
IGHV13-2*03	TATTGTAGCAGA		(F)
IGHV14-1*01	TACTGTACTACA		F

IGHV14-1*02	TACTGTGCTAGA	CTAGA	F
IGHV14-2*01	TACTGTGCTAGA	CTAGA	F
IGHV14-2*02	TACTGTGCTAGA	CTAGA	P
IGHV14-3*01	TACTGTGCTAGA	CTAGA	F
IGHV14-3*02	TACTGTGCTAGA	CTAGA	F
IGHV14-4*01	TACTGTACTACA		F
IGHV14-4*02	TACTGTAATGCA		F
IGHV14S4*01	TACTGTCCCTAT		(F)
IGHV15-2*01	TACTGTGCAAGG	CAAGG	F
IGHV15-2*02	TACTGTGCAAGG	CAAGG	(F)
IGHV16-1*01	TATTGTGCCAGAGA		F
IGHV2-2*01	TACTGTGCCAGAAA	CCAGAAA	F
IGHV2-2*02	TACTGTGCCAGAAA	CCAGAAA	F
IGHV2-2*03	TACTGTGCCAGAAA	CCAGAAA	F
IGHV2-2-1*01	TACTGTGCCAGAAA	CCAGAAA	P
IGHV2-2-2*01	TACTGTGTCAGAAA	TCAGAAA	F
IGHV2-3*01	TACTGTGCCAAACC	CCAAACC	F
IGHV2-3-1*01	TACTGTGCCAGAAA	CCAGAAA	F
IGHV2-4*01	TACTGTGCCAAAAA	CCAAAAA	F
IGHV2-4*02	TACTGTGCCAGAAA	CCAGAAA	F
IGHV2-4-1*01	TACTGTGCCAGAAA	CCAGAAA	F
IGHV2-5*01	TACTGTGCCAAAAA	CCAAAAA	F
IGHV2-5-1*01	TACTGTGCCAAAAA	CCAAAAA	F
IGHV2-6*01	TACTGTGCCAGTG	CCAGTG	F
IGHV2-6*02	TACTGTGCCAGAAA	CCAGAAA	F
IGHV2-6*03	TACTGTGCCAGA	CCAGA	F
IGHV2-6-1*01	TACTGTGCCAGACA	CCAGACA	F
IGHV2-6-2*01	TACTGTGCCAGACA	CCAGACA	F
IGHV2-6-3*01	TACTGTGTAAGAGA	TAAGAGA	F
IGHV2-6-4*01	TACTGTGCCAGAAA	CCAGAAA	F
IGHV2-6-5*01	TACTGTGCCAAACA	CCAAACA	F
IGHV2-6-6*01	TACTGTGCCAAACC	CCAAACC	F
IGHV2-6-7*01	TACTGTGCCAGAGA	CCAGAGA	F
IGHV2-6-7*02	TACTGTGCCAGAGA	CCAGAGA	F
IGHV2-6-8*01	TACTGTGCCAGTGA	CCAGTGA	F
IGHV2-7*01	CACTGTGCCAGATA		F
IGHV2-9*01	TACTGTGCACAAAC	CACAAAC	F
IGHV2-9*02	TACTGTGTAAGAGA	TAAGAGA	F
IGHV2-9-1*01	TACTGTGCCAGAGA	CCAGAGA	F
IGHV2S3*01	TACTGTGTAAGAGA	TAAGAGA	F
IGHV3-1*01	TACTGTGCAAGAAGGA	CAAGAAGGA	F
IGHV3-1*02	TACTGTGCAAGA	CAAGA	F
IGHV3-2*02	TACTGTGCAAGA	CAAGA	F
IGHV3-3*01	TACTGTGCGAGAGA	CGAGAGA	F
IGHV3-3*02	TACTGTGCGAGAGA	CGAGAGA	P
IGHV3-3*03	TACTGTGCGAGAGA	CGAGAGA	F
IGHV3-4*01	TACTGTGCAAGAGA	CAAGAGA	F
IGHV3-4*02	TACTGTGCAAGAGA	CAAGAGA	F
IGHV3-5*01	TACTGTGCACGAGA	CACGAGA	F
IGHV3-5*02	TACTGTGCACGAGA	CACGAGA	F
IGHV3-6*01	TACTGTGCAAGAGA	CAAGAGA	F
IGHV3-6*02	TACTGTGCAAGAGA	CAAGAGA	F
IGHV3-6*03	TACTGTGCACGA	CACGA	(F)
IGHV3-7*01	TACTGTGCAAACAG	CAAACAG	P
IGHV3-7*02	TACTGTGCAAATGA	CAAATGA	P
IGHV3-8*01	TACTGTGCAAGAAT	CAAGAAT	F
IGHV3-8*02	TACTGTGCAAGATA	CAAGATA	F

IGHV3S1*01	TACTGTGCAAGATA	CAAGATA	F
IGHV3S1*02	TACTGTGCAAGATA	CAAGATA	F
IGHV3S7*01	TACTGTGCAAGAGG	CAAGAGG	(F)
IGHV4-1*01	TACTGTGCAAGA	CAAGA	F
IGHV4-1*02	TACTGTGCAAGACC	CAAGACC	F
IGHV4-2*01	TACTGTGCAAGACC	CAAGACC	P
IGHV4-2*02	TACTGTGCAAGACT	CAAGACT	F
IGHV5-1*01	TACTGTTTGAGAAC		P
IGHV5-1*02	TACTGTTTGAGACA		P
IGHV5-12*01	TACTGTGCAAGAAC	CAAGAAC	F
IGHV5-12*02	TACTGTGCAAGACA	CAAGACA	F
IGHV5-12*03	TACTGTGCAAGACGA	CAAGACGA	[F]
IGHV5-12-1*01	TACTGTGCAAGACA	CAAGACA	F
IGHV5-12-2*01	TACTGTGCAAGACA	CAAGACA	F
IGHV5-12-2*02	TACTGTGCAAGACA	CAAGACA	F
IGHV5-12-4*01	TACTGTGCAAGA	CAAGA	F
IGHV5-15*01	TACTGTGCAAGAAC	CAAGAAC	F
IGHV5-15*02	TACTGTGCAAGGGA	CAAGGGA	F
IGHV5-15*03	TACTGTGCAAGA	CAAGA	[F]
IGHV5-15*04	TACTGTGCAAGACGA	CAAGACGA	[F]
IGHV5-15*05	TACTGTGCAAGACA	CAAGACA	F
IGHV5-16*01	TACTGTGCAAGAAG	CAAGAAG	F
IGHV5-16*02	TACTGTGCAAGACGA	CAAGACGA	[F]
IGHV5-17*01	TACTGTGCAAGG	CAAGG	F
IGHV5-17*02	TACTGTGCAAGA	CAAGA	F
IGHV5-17*03	TACTGTGCAAGACGA	CAAGACGA	[F]
IGHV5-2*01	TACTGTGCAAGAAC	CAAGAAC	F
IGHV5-2*02	TACTGTGCAAGACA	CAAGACA	F
IGHV5-2*03	TACTGTGCAAGACGA	CAAGACGA	[F]
IGHV5-21*01	TACTGTGCAAGAG	CAAGAG	P
IGHV5-4*01	TACTGTGCAAGAAG	CAAGAAG	F
IGHV5-4*02	TACTGTGCAAGAGA	CAAGAGA	F
IGHV5-4*03	TACTGTGCAAGACGA	CAAGACGA	[F]
IGHV5-6*01	TACTGTGCAAGACA	CAAGACA	F
IGHV5-6*02	TACTGTGCAAGACGA	CAAGACGA	[F]
IGHV5-6*03	TACTGTGCAAGACGA	CAAGACGA	[F]
IGHV5-6-1*01	TACTGTGCAAGACA	CAAGACA	F
IGHV5-6-2*01	TACTGTGCAAGACA	CAAGACA	F
IGHV5-6-2*02	TACTGTGCAAGA	CAAGA	F
IGHV5-6-3*01	TACTGTGCAAGAGA	CAAGAGA	F
IGHV5-6-3*02	TACTGTGCAAGAGA	CAAGAGA	F
IGHV5-6-4*01	TACTGTACAAGAGA		F
IGHV5-6-4*02	TACTGTACAAGA		F
IGHV5-6-5*01			F
IGHV5-6-6*01	TACTGTGCAAGA	CAAGA	F
IGHV5-9*01	TACTGTGCAAGAAC	CAAGAAC	F
IGHV5-9*02	TACTGTGCAAGACA	CAAGACA	F
IGHV5-9*03	TACTGTGCAAGATA	CAAGATA	F
IGHV5-9*04	TACTGTGCAAGAC	CAAGAC	[F]
IGHV5-9-1*01	TACTGTGCAAGA	CAAGA	F
IGHV5-9-1*02	TACTGTACAAGA		F
IGHV5-9-2*01	TACTGTGCAAGACA	CAAGACA	F
IGHV5-9-3*01	TACTGTGCAAGACA	CAAGACA	F
IGHV5-9-4*01	TACTGTGCAAGGGA	CAAGGGA	F
IGHV5-9-5*01	TACTGTGCAAGATA	CAAGATA	F
IGHV5S12*01	TACTGTGCAAGA	CAAGA	[F]
IGHV5S21*01	TACTGTGCAAGACGA	CAAGACGA	[F]

IGHV5S24*01	TACTGTGCAAGACGA	CAAGACGA	[F]
IGHV5S4*01	TACTGTGCAAGA	CAAGA	F
IGHV5S4*02	TACTGTGCAAGACA	CAAGACA	F
IGHV5S9*01	TACTGTGCAAGACA	CAAGACA	F
IGHV6-3*01	TACTGCACAGG		F
IGHV6-3*02	TACTGCACAGG		F
IGHV6-3*03	TACTGCACAGGC		F
IGHV6-4*01	TACTGTACAAGG		F
IGHV6-4*02	TACTGTACAAAGG		F
IGHV6-5*01	TACTGTACAAGG		F
IGHV6-5*02	TACTGTAAAGG		F
IGHV6-6*01	TACTGTACCAGG		F
IGHV6-6*02	TACTGTACCAGG		F
IGHV6-7*01	TACTGTACATGG		F
IGHV6-7*02	TACTGTACATGG		F
IGHV6S2*01			[F]
IGHV6S3*01			[F]
IGHV6S4*01			[F]
IGHV7-1*01	TACTGTGCAAGAACGTAG	CAAGAACGTAG	P
IGHV7-1*02	TACTGTGCAAGAGATGCA	CAAGAGATGCA	F
IGHV7-1*03	TACTGTGCAAGAGATGCA	CAAGAGATGCA	F
IGHV7-2*01	TACTGTGCAAGAGATACA	CAAGAGATACA	F
IGHV7-3*01	TACTGTGCAAGATATA	CAAGATATA	F
IGHV7-3*02	TACTGTGCAAGAGATA	CAAGAGATA	F
IGHV7-3*03	TACTGTGCAAAAGATA	CAAAAGATA	F
IGHV7-3*04	TACTGTGCAAGAGATA	CAAGAGATA	F
IGHV7-4*01	TACTGTGTAAAAGCTGTA	TAAAAGCTGTA	F
IGHV7-4*02	TACTGTGCAAAAGATGTA	CAAAAGATGTA	F
IGHV7-4*03	TACTGTGCAAAA	CAAAA	F
IGHV7-4*04	TACTGTGTAAAAGCTGTA	TAAAAGCTGTA	F
IGHV8-2*01	TACGGTGCTTGGAGAGAG		ORF
IGHV8-4*01	TACTGTGCT	CT	F
IGHV8-5*01	TACTGTGCTCAAATAG	CTCAAATAG	F
IGHV8-5*02			[F]
IGHV8-6*01	TACTGTGCTCGA	CTCGA	F
IGHV8-7*01	TACTGTGCT	CT	P
IGHV8-8*01	TACTGTGCTCGAATA	CTCGAATA	F
IGHV8-8*02			[F]
IGHV8-8-1*01	TACTGTGCTCACAGAC	CTCACAGAC	ORF
IGHV8-9*01	TACTCTGCTCGAAGAG		ORF
IGHV8-9*02	TGA		[P]
IGHV8-9*03			[F]
IGHV8-10*01	TACTGTGCTTGAGGAG	CTTGAGGAG	P
IGHV8-11*01	TACTGTGCTCGAATAG	CTCGAATAG	F
IGHV8-12*01	TACTGTGCTCGAAGAG	CTCGAAGAG	F
IGHV8-13*01	TACTGTGCTCGAAG	CTCGAAG	ORF
IGHV8-14*01	TACTGTGCTTGAGGAG	CTTGAGGAG	P
IGHV8S2*01	TACTGTGCTTGAGGAG	CTTGAGGAG	P
IGHV8S6*01			[P]
IGHV8S9*01			[F]
IGHV9-1*01	TTCTGTGTAAGA		F
IGHV9-1*02	TTCTGTGCAAGA		F
IGHV9-1*03			F
IGHV9-1*04			F
IGHV9-2*01	TTCTGTGCAAGA		F
IGHV9-2*02	TTCTGTGCAAGA		F
IGHV9-2-1*01	TTCTGTGCTAGA		F

IGHV9-3*01	TTCTGTGCAAGA	F
IGHV9-3*02	TTCTGTGCAAGA	F
IGHV9-3*03	TTCTGT	F
IGHV9-3-1*01	TTCTGTGCAAGA	F
IGHV9-4*01	TTCTGTGCGAGAA	F
IGHV9-4*02	TTCTGTGCGAGA	F
IGHV9-4*03	TTCTGT	F
IGHV9S7*01	TTCTGT	F
IGHV9S8*01	TTCTGT	F

Note: F, functional; P, pseudogene; ORF, open reading frame; noFuncSign, signed as non-functional.

Supplemental Table 2a. Potential Human IgH V_H Replacement Footprints.

minimal footprint length(#)	10(1)	9(4)	8(7)	7(25)	6(40)	5(45)	4(41)	3(29)
possible footprints	CACACAGACC	CACGGATAC	CACGGATA	CGAGAGA	CGAGAG	CGAGA	CGAG	CGA
		CACACAGAC	ACGGATAC	CAACAGA	GAGAGA	GAGAG	GAGA	GAG
		ACACAGACC	CACACAGA	CAAGANA	CAACAG	AGAGA	AGAG	AGA
		CAAAAGATA	ACACAGAC	CAAGATA	AACAGA	CAACA	CAAC	CAA
			CACAGACC	CTAGAGA	CAAGAN	AACAG	AACA	AAC
			CAAAAGAT	CGGCAGA	AAGANA	ACAGA	ACAG	ACA
			AAAAGATA	CGAGAGG	CAAGAT	CAAGA	CAGA	CAG
				CACGGAT	AAGATA	AAGAN	CAAG	AAG
				ACGGATA	CTAGAG	AGANA	AAGA	GAN
				CGGATAC	TAGAGA	AAGAT	AGAN	ANA
				CACACAG	CGGCAG	AGATA	GANA	GAT
				ACACAGA	GGCAGA	CTAGA	AGAT	ATA
				CACAGAC	GAGAGG	TAGAG	GATA	CTA
				ACAGACC	CACGGA	CGGCA	CTAG	TAG
				CAAGAGA	ACGGAT	GGCAG	TAGA	CGG
				CGAAAGA	CGGATA	GCAGA	CGGC	GGC
				CAAAAGA	GGATAC	AGAGG	GGCA	GCA
				AAAAGAT	CACACA	CACGG	GCAG	AGG
				AAAGATA	ACACAG	ACGGA	GAGG	CAC
				CTAGGGA	CACAGA	CGGAT	CACG	ACG
				TGAAAGA	ACAGAC	GGATA	ACGG	GGA
				CGAGACA	CAGACC	GATAC	CGGA	TAC
				CGAGAAA	CAAGAG	CACAC	GGAT	GAC
				CCAGAGA	AAGAGA	ACACA	ATAC	ACC
				CGAGATA	CGAAAG	CACAG	CACA	GAA
					GAAAGA	CAGAC	ACAC	AAA
					CAAAAG	AGACC	AGAC	GGG
					AAAAGA	TACGG	GACC	TGA
					AAAGAT	AAGAG	TACG	CCA
					CTAGGG	CGAAA	CGAA	
					TAGGGA	GAAAG	GAAA	
					TGAAAG	AAAGA	AAAG	
					CGAGAC	CAAAA	CAAA	
					GAGACA	AAAAG	AAAA	
					CGAGAA	CTAGG	TAGG	
					GAGAAA	TAGGG	AGGG	
					CCAGAG	AGGGA	GGGA	
					CAGAGA	TGAAA	TGAA	
					CGAGAT	GAGAC	GACA	
					GAGATA	AGACA	AGAA	
						GAGAA	CCAG	
						AGAAA		
						CCAGA		
						CAGAG		
						GAGAT		

Supplemental Table 2b. Potential Mouse IgH V_H Replacement Footprints.

minimal footprint length(#)	11(6)	10(12)	9(25)	8(36)	7(68)	6(85)	5(84)	4(72)	3(42)
possible footprints	CAGGAAGACAG	CAGGAAGACA	CAGGAAGAC	CAGGAAGA	TGAGACA	TGAGAC	TGAGA	TGAG	TGA
	CAGGAGACAGA	AGGAAGACAG	AGGAAGACA	AGGAAGAC	TGAGCGA	GAGACA	GAGAC	GAGA	GAG
	CAAGAGATGCA	CAGGAGACAG	GGAAGACAG	GGAAGACA	TGAGAGA	TGAGCG	AGACA	AGAC	AGA
	CAAGAGATACA	AGGAGACAGA	CAGGAGACA	GAAGACAG	GAAGAGG	GAGCGA	TGAGC	GACA	GAC
	TAAAAGCTGTA	CAAGAGATGC	AGGAGACAG	CAGGAGAC	CAGGAAG	TGAGAG	GAGCG	GAGC	ACA
	CAAAAGATGTA	AAGAGATGCA	GGAGACAGA	AGGAGACA	AGGAAGA	GAGAGA	AGCGA	AGCG	AGC
		CAAGAGATAC	CAAGAGAGA	GGAGACAG	GGAAGAC	GAAGAG	GAGAG	GCGA	GCG
		AAGAGATACA	CAAGAAGGA	GAGACAGA	GAAGACA	AAGAGG	AGAGA	AGAG	CGA
		TAAAAGCTGT	CAAGAGATG	CAAGAGAG	AAGACAG	CAGGAA	GAAGA	GAAG	GAA
		AAAAGCTGTA	AAGAGATGC	AAGAGAGA	CAGGAGA	AGGAAG	AAGAG	AAGA	AAG
		CAAAAGATGT	AGAGATGCA	CAAGAAGG	AGGAGAC	GGAAGA	AGAGG	GAGG	AGG
		AAAAGATGTA	CAAGAGATA	AAGAAGGA	GGAGACA	GAAGAC	CAAGA	CAAG	CAA
			AAGAGATAC	CAAGAGAT	GAGACAG	AAGACA	CAGGA	CAGG	CAG
			AGAGATACA	AAGAGATG	AGACAGA	AGACAG	AGGAA	AGGA	GGA
			CAAGATATA	AGAGATGC	CAAGAGA	CAGGAG	GGAAG	GGAA	CTA
			CAAAAGATA	GAGATGCA	AAGAGAG	AGGAGA	AAGAC	ACAG	TAG
			TAAAAGCTG	AAGAGATA	AGAGAGA	GGAGAC	GACAG	GGAG	AAT
			AAAAGCTGT	AGAGATAC	CCAGAAA	GACAGA	AGGAG	CAGA	ATA
			AAAGCTGTA	GAGATACA	TCAGAAA	CAAGAG	GGAGA	CTAG	CCA
			CAAAAGATG	CAAGATAT	CCAAACC	AAGAGA	ACAGA	TAGA	AAA
			AAAAGATGT	AAGATATA	CCAAAAA	AGAGAG	CTAGA	AAGG	TCA
			AAAGATGTA	CAAAAGAT	CCAGACA	CCAGAA	CAAGG	CAAT	AAC
			CTCGAATAG	AAAAGATA	TAAGAGA	CAGAAA	CAATA	AATA	ACC
			CTCGAAGAG	TAAAAGCT	CCAAACA	TCAGAA	CCAGA	CCAG	AGT
			CTCAAATAG	AAAAGCTG	CCAGAGA	CCAAAC	CAGAA	AGAA	GTG
				AAAGCTGT	CCAGTGA	CAAACC	AGAAA	GAAA	TAA
				AAGCTGTA	CACAAAC	CCAAAA	TCAGA	TCAG	CAC
				AAAAGATG	CAAGAAG	CAAAAA	CCAAA	CCAA	ACG
				AAAGATGT	AAGAAGG	CCAGTG	CAAAC	CAAA	GAT
				AAGATGTA	AGAAGGA	CCAGAC	AAACC	AAAC	ACT
				CTCGAATA	CGAGAGA	CAGACA	CAAAA	AACC	GGG
				TCGAATAG	CACGAGA	TAAGAG	AAAAA	AAAA	ATG
				CTCGAAGA	CAAGAAT	CAAACA	CCAGT	CAGT	TGC
				TCGAAGAG	CAAGATA	CCAGAG	CAGTG	AGTG	GCA
				CTCAAATA	CAAGACC	CAGAGA	CAGAC	TAAG	TAC
				TCAAATAG	CAAGACT	CAGTGA	TAAGA	AACA	TAT
					CAAGAAC	CACAAA	AAACA	GTGA	GCT
					CAAGACA	ACAAAC	CAGAG	CACA	CTG
					CAAGGGA	CAAGAA	AGTGA	ACAA	TGT
					AAGAGAT	AAGAAG	CACAA	CGAG	GTA
					AGAGATG	AGAAGG	ACAAA	CACG	CTC
					GAGATGC	GAAGGA	AAGAA	ACGA	TCG
					AGATGCA	CGAGAG	AGAAG	GAAT	
					AGAGATA	CACGAG	GAAGG	AGAT	
					GAGATAC	ACGAGA	AAGGA	GATA	
					AGATACA	AAGAAT	CGAGA	GACC	
					AAGATAT	CAAGAT	CACGA	GACT	
					AGATATA	AAGATA	ACGAG	GAAC	
					CAAAAGA	CAAGAC	AGAAT	AGGG	
					AAAAGAT	AAGACC	AAGAT	GGGA	
					AAAGATA	AAGACT	AGATA	GATG	
					TAAAAGC	AAGAAC	AGACC	ATGC	
					AAAAGCT	CAAGGG	AGACT	TGCA	

AAAGCTG	AAGGGA	AGAAC	ATAC
AAGCTGT	AGAGAT	AAGGG	TACA
AGCTGTA	GAGATG	AGGGA	ATAT
AAAGATG	AGATGC	GAGAT	TATA
AAGATGT	GATGCA	AGATG	AAAG
AGATGTA	GAGATA	GATGC	TAAA
CTCGAAT	AGATAC	ATGCA	AAGC
TCGAATA	GATACA	GATAC	AGCT
CGAATAG	AGATAT	ATACA	GCTG
CTCGAAG	GATATA	GATAT	CTGT
TCGAAGA	CAAAAG	ATATA	TGTA
CGAAGAG	AAAAGA	AAAAG	ATGT
CTCAAAT	AAAGAT	AAAGA	CTCG
TCAAATA	TAAAAG	TAAAA	TCGA
CAAATAG	AAAAGC	AAAGC	CGAA
	AAAGCT	AAGCT	ATAG
	AAGCTG	AGCTG	CTCA
	AGCTGT	GCTGT	TCAA
	GCTGTA	CTGTA	AAAT
	AAGATG	GATGT	
	AGATGT	ATGTA	
	GATGTA	CTCGA	
	CTCGAA	TCGAA	
	TCGAAT	CGAAT	
	CGAATA	GAATA	
	GAATAG	AATAG	
	TCGAAG	CGAAG	
	CGAAGA	CTCAA	
	CTCAAA	TCAAA	
	TCAAAT	CAAAT	
	CAAATA	AAATA	
	AAATAG		
